# Housing and personality effects on judgement and attention biases in dairy cows

**DOI:** 10.1038/s41598-021-01843-w

**Published:** 2021-11-26

**Authors:** Louise Kremer, Jacinta D. Bus, Laura E. Webb, Eddie A. M. Bokkers, Bas Engel, Jozef T. N. van der Werf, Sabine K. Schnabel, Cornelis G. van Reenen

**Affiliations:** 1 Animal Production Systems Group, Wageningen University & Research, PO Box 338, 6700 AH Wageningen, The Netherlands; 2 Livestock Research, Wageningen University & Research, PO Box 338, 6700 AH Wageningen, The Netherlands; 3 Biometris, Wageningen University & Research, PO Box 16, 6700 AA Wageningen, The Netherlands

**Keywords:** Animal behaviour, Attention, Decision, Personality

## Abstract

Affective states can be inferred from responses to ambiguous and threatening stimuli, using Judgement Bias Tasks (JBTs) and Attention Bias Tasks (ABTs). We investigated the separate and interactive effects of personality and housing conditions on dairy cattle affective states. We assessed personality in 48 heifers using Open-Field, Novel-Object and Runway tests. Personality effects on responses to the JBT and to the ABT were examined when heifers were housed under reference conditions. Heifers were subsequently housed under positive or negative conditions, and housing effects on animal responses in both tasks were investigated while controlling for personality. A Principal Component Analysis revealed three personality traits labelled Activity, Fearfulness and Sociability. Under reference conditions, personality influenced heifers’ responses to the JBT and to the ABT, therefore questioning the tasks’ generalizability across individuals. Against expectations, housing did not influence responses to the  JBT and heifers in the negative conditions looked at the threat later than heifers in the positive or reference conditions. More research is warranted to confirm the validity and the repeatability of the JBT and of the ABT as appropriate measures of affective states in dairy cows.

## Introduction

The welfare of dairy cows is a major societal concern^1^, insomuch that consumers are willing to pay more for products obtained from animals whose welfare has not been compromised^2^. The concept of animal welfare initially revolved around major threats to animal survival (e.g. disease, thirst…), but it progressively evolved as research progressed and public values changed^3^. Nowadays, the definition of animal welfare includes the notion of affective states^4,5^, which reflects the animal's subjective experience of events. Animal welfare is now considered optimal when the balance between positive and negative affective states is overall positive^6^. Adequate evaluation of cow welfare, therefore, requires valid measures of cow affective states^7–9^. Several methodologies have been developed to this end, based on research in human cognitive psychology^10^.

Animal affective states can be inferred from biases in cognition^11^. Two cognitive biases, the judgement and attention biases, have been assessed in farm animals using standardised paradigms^12–15^. Judgement biases, which reflect affect-driven shifts in the interpretation of ambiguous stimuli^16^, are assessed using Judgement Bias Tasks (JBTs)^17^. The JBT principle relies on the idea that animals in positive affective states judge ambiguous situations more positively (i.e. more optimistically) than animals in negative affective states—and vice versa^17^. Attention biases, which reflect affect-driven shifts in the allocation of attention to salient stimuli^16^, are assessed using Attention Bias Tasks (ABTs)^18^. The ABT principle relies on the idea that attention to threat is influenced by one's affective states^19^. Cows and sheep, for instance, have heightened attention to threatening stimuli when in heightened anxious states^14,15^. With JBTs and ABTs, researchers can investigate the effects of various treatments on animal affective states, by assessing changes in pessimism^20,21^ and in attention to threat^22–24^. Two main sources of variation in judgement and attention biases have commonly been explored, namely the living environment and animal personality.

The living environment influences animal affective states, and subsequent affect-driven cognitive biases, by conditioning individuals’ propensity to experience positive and negative events. For captive animals, the housing conditions constitute the main aspect of their living environment. Previous studies have investigated the effects of housing conditions on cognitive biases in different farm species^25,26^, with a predominant focus on judgement bias. For instance, pigs housed in enriched conditions seemed more optimistic than those housed in barren conditions^25^. Similarly, pair-housed calves seemed more optimistic than individually-housed calves^27^. Recently, one study also revealed that housing conditions influenced attention biases in pigs^22^. Negative and positive housing contrasts may, therefore, constitute promising models of animal affective states.

Beside housing conditions, personality may also modulate animal judgement and attention biases. Personality traits—defined as a correlated set of individual behavioural and physiological traits that are consistent over time and across contexts^28,29^—may influence animal affective states by mediating subjective experiences of events^30^. For instance, calves characterised as ‘fearful’, i.e. relatively slow at reaching novel-objects and unfamiliar humans, were more pessimistic than non-fearful calves, while housed under the same conditions^31^. Similarly, parrots characterised as neurotic, i.e. relatively excitable, fearful and non-social, had greater attention bias to an unfamiliar human than non-neurotic parrots, while housed under the same conditions^32^. Accounting for individual variations, and in particular for personality differences, appears hence necessary to validate the use of housing contrasts as model of affective states.

Furthermore, housing and personality may exert an interactive effect on animal judgement and attention biases. Several studies revealed that housing-induced judgement biases^33,34^ and attention biases^22^ are dependent upon animal personality traits. For instance, Ross and colleagues^34^ reported that the level of enrichment in the housing conditions of hens influenced the judgement bias responses of individuals characterised as exploratory—i.e. hens approaching a novel object relatively fast—but not the responses of individuals characterised as non-exploratory. Depending on their personality, certain sub-populations of animals may hence be more sensitive than others to specific housing conditions.

Therefore, in our study, we aimed at investigating:

1. The effect of personality traits on judgement and attention biases in dairy heifers kept under reference housing conditions (i.e. at baseline) to minimize variations in individual affective background.
2. The effect of (supposedly) affectively-contrasted housing conditions on heifer judgement and attention biases by using a longitudinal approach to control for individual variation.
3. Individual variation consistency in heifer responses to the cognitive bias tests across (supposedly) contrasted housing conditions—either by focusing on untargeted sources of individual variation or by tentatively exploring the effects of targeted sources of individual variation, i.e. identified personality traits, on dairy heifers’ cognitive bias responses.

## Results

### Identification of dairy heifers' personality traits

Behavioural data from the personality tests of 47 heifers were suitable for PCA analysis, as reflected by the overall Kaiser–Meyer–Olkin criterion (= 0.71) and each variable's Kaiser–Meyer–Olkin criterion (from 0.48 to 0.77). The hypothesis of all zero correlation was rejected (Bartless’s sphericity test, p < 0.001). The first three Rotated Components (RCs) explained 80% of the total variance. Loadings on the first three RCs are presented in Table 1. Heifers scoring high on RC1 explored and walked the most in the arena. Those scoring high on RC2 spent the most time in contact with the Novel Object (NO) and were the fastest to reach the NO for the first time. Finally, heifers scoring high on RC3 spent more time within the 2 m zone, i.e. close to other cows, in the runway (RW) test. For ease of reading, RCs are hereafter referred to as “personality traits”. RC1 is labelled the Activity trait, RC2 the Fearfulness trait and RC3 the Sociability trait. The repartition of heifers per different personality trait and within each type of housing conditions is detailed in Table 2.

**Table 1:** Loadings of the behavioural measures on the 3 main rotated components (RCs).

Behavioural measures	RC1	RC2	RC3
Number of locomotion bouts in OF and NO	**0.817**	0.314	− 0.079
Time spent in locomotion in OF and NO	**0.854**	0.153	0.114
Time spent in contact with walls/floor in OF and NO	**0.843**	0.071	− 0.068
Time spent in contact with NO	0.120	**0.886**	− 0.128
Latency to touch NO	− 0.241	**− 0.841**	− 0.012
Time spent within 2 m from the group in RW	− 0.011	− 0.085	**0.990**
Eigenvalue	2.72	1.19	0.91

Loadings rated ‘excellent' (i.e. (|values| > 0.71) are written in bold.

*NO* Novel-object, *OF* Open-field, *RW* Runway.

**Table 2:** Number of heifers in each housing conditions per personality trait.

Personality			Housing conditions (number)		
Trait (median score)	Class	Definition	Reference (n = n_1_ + n_2_)	Positive (n_1_)	Negative (n_2_)
RC1: Activity (− 0.08)	Active	Sup. to − 0.08	23	11	12
	Inactive	Inf. or equal to − 0.08	24	13	11
RC2: Fearfulness (0.23)	Fearful	Inf. or equal to 0.23	24	13	11
	Non-fearful	Sup. to 0.23	23	11	12
RC3: Sociability (0.26)	Social	Sup. to 0.26	24	10	14
	Non-social	Inf. or equal to 0.26	23	14	9

Heifers were divided in two classes per personality trait based on their behavioural scores on the related personality trait in comparison to the median score. *Sup*. Superior to, *Inf.* Inferior to, *RC* rotated component, *n* number of heifers per personality class in the reference conditions, *n*_*1*_ number of heifers per personality class in the positive conditions, *n*_*2*_ number of heifers per personality class in the negative conditions.

### Personality effect on heifers’ responses to judgement and attention bia s tests under the reference conditions

The main effect of personality on individual responses to the cognitive bias tasks was assessed in the reference conditions only, when variations in individual affective background were expected to be minimal. Regarding the JBT results, there was a significant interaction of Activity and Fearfulness on heifers’ *Average latency to reach the ambiguous cues* (p = 0.001). In particular, inactive fearful heifers were slower to reach the ambiguous cues (i.e. more pessimistic) than inactive non-fearful heifers (inactive fearful: 73% ± 8.7, inactive non-fearful: 33% ± 8.8, p = 0.032). Sociability had no significant effect on latency to reach the cues (p = 0.150).

Regarding the ABT results, there was a significant interaction between Fearfulness and Sociability on *Time spent eating* (p = 0.018). Non-fearful non-social heifers spent more time eating (46% ± 13.8) than fearful non-social heifers (9% ± 5.1, p < 0.001), fearful social heifers (9% ± 5.0, p < 0.001) and non-fearful social heifers (26% ± 9.0, p = 0.027). The effect of personality on the behaviours observed under the reference conditions during the ABT are detailed in Table 3.

**Table 3:** Behavioural measures obtained in the Attention Bias Task under the reference conditions.

Personality		Latency to look at the threat		Latency to eat from the bucket			
Trait	Class	Mean (%) ± s.e.m	p-value	Mean (%) ± s.e.m	p-value		
Activity	Active	2 ± 0.6	0.500	57 ± 8.4	0.410		
	Inactive	2 ± 0.5		58 ± 9.5			
Fearfulness	Fearful	2 ± 0.4	0.990	64 ± 8.2	0.260		
	Non-fearful	2 ± 0.7		51 ± 9.4			
Sociability	Social	**2 ± 0.4**	**0.043**	63 ± 8.3	0.130		
	Non-social	**3 ± 0.7**		51 ± 9.4			

Personality		Time spent looking at the threat		Time spent eating		Relative positive attention	
Trait	Class	Mean (%) ± s.e.m	p-value	Mean (%) ± s.e.m	p-value	Mean (%) ± s.e.m	p-value
Activity	Active	13 ± 2.6	0.330	**20 ± 5.8**	**0.013**	53 ± 6.9	0.360
	Inactive	14 ± 3.2		**23 ± 7.4**		52 ± 8.8	
Fearfulness	Fearful	**17 ± 3.3**	**0.039**	9 ± 3.5	NA^a^	**41 ± 6.4**	**0.023**
	Non-fearful	**10 ± 1.9**		34 ± 7.8		**64 ± 8.3**	
Sociability	Social	13 ± 1.9	0.970	19 ± 5.7	NA^a^	52 ± 7.5	0.560
	Non-social	14 ± 3.7		24 ± 7.4		53 ± 8.1	

Personality		Head up		In locomotion		In contact with walls/floor	
Trait	Class	Mean (%) ± s.e.m	p-value	Mean (%) ± s.e.m	p-value	Mean (%) ± s.e.m	p-value
Activity	Active	24 ± 4.9	0.670	**27 ± 3.3**	**0.026**	15 ± 2.0	0.052
	Inactive	28 ± 5.3		**17 ± 2.0**		11 ± 2.7	
Fearfulness	Fearful	32 ± 4.8	0.098	**27 ± 3.2**	**0.013**	12 ± 2.0	0.810
	Non-fearful	19 ± 4.9		**18 ± 2.5**		14 ± 2.7	
Sociability	Social	19 ± 3.5	0.089	22 ± 3.0	0.600	**16 ± 2.5**	**0.013**
	Non-social	33 ± 6.0		23 ± 3.1		**10 ± 2.0**	

Results are presented according to the personality traits and classes of personality trait. Results are expressed in proportion of trial duration (120 s), except for *Relative positive attention,* which is expressed in proportion of heifer’s total time spent at looking at the stimuli. Significant results are written in bold. ^a^NA (not applicable) instead of an exact p-value is indicated in case of interaction effects between personality traits.

### Housing effects on heifers’ responses to the judgement and attention bias tests

The longitudinal analyses of cognitive biases *both* in the reference and in the experimental conditions were used to assess the main effect of housing on heifers’ cognitive biases while controlling for individual variations—including personality differences. Regarding the JBT results, housing did not influence heifers’ *Average latency to reach the ambiguous cues* (p = 0.700, reference: 52% of total trial duration ± 5.0, positive: 54% ± 6.4, negative 59% ± 6.9).

Regarding the ABT results, heifers in the negative conditions looked at the threat later and walked less than heifers in the positive conditions (p < 0.001 and p = 0.011, respectively). Furthermore, heifers in the positive, but not negative, conditions spent less time looking at the threat than they did during the reference conditions (p = 0.005). Housing effects on heifers’ behaviours during the ABT are presented in Table 4. Behavioural responses obtained for each housing condition are detailed according to personality traits, personality classes and housing conditions in Supplementary Tables 2–4 online.

**Table 4:** Average ± s.e.m. of each behavioural response observed during the Attention Bias Tasks according to the housing conditions.

Response variables	Reference	Positive	Negative
Latency to look at the threat	2 ± 0.4^a^	1 ± 0.3^a^	7 ± 2.4^b^
Latency to eat	54 ± 6.5^a^	36 ± 8.6^b^	31 ± 9.1^b^
Time spent looking at the threat	13 ± 2.2^a^	6 ± 1.5^b^	9 ± 3.2^ab^
Time spent eating	22 ± 4.6^a^	36 ± 7.2^b^	49 ± 8.3^b^
Relative positive attention	55 ± 5.7^a^	78 ± 5.6^b^	78 ± 8.1^b^
Time spent in locomotion	22 ± 2.1^a^	18 ± 2.3^a^	13 ± 2.0^b^
Time spent in contact with walls	13 ± 1.8^a^	13 ± 3.3^a^	10 ± 2.3^a^
Time spent with head up	25 ± 3.7^a^	20 ± 5.2^a^	11 ± 3.3^a^

Different letters indicate statistical differences between the housing conditions and were extracted from post-hoc testing (p < 0.05 after Bonferroni correction).

### Relationships between cognitive bias responses in the reference and in the experimental conditions

The analyses of covariance allowed for the assessment of consistency in heifers’ judgement and attention biases across the different housing conditions, which is used as a measure of unspecific personality influences on heifers’ responses to the cognitive bias tests. Regarding the JBT results**,** there was a positive linear relationship between *Average latency to reach the ambiguous cues* in the reference and in the experimental conditions (ß = 0.168, p = 0.034). There was no evidence of a housing effect on *Average latency to reach the ambiguous cues* in the experimental conditions when controlling for individual response in the reference conditions (p = 0.660).

Regarding the ABT results, there was an interaction effect between the covariate *Time spent with head up* in the reference conditions and housing on *Time spent with head up* in the experimental conditions (p = 0.007). We found a negative linear relationship between *Time spent with head up* in the reference conditions and in the positive housing conditions, but not between *Time spent with the head up* in the reference conditions and in the negative housing conditions. Results from the ANCOVAs are presented in Table 5.

**Table 5:** Regression coefficients (ß), standard errors (in brackets) and p-values of the behavioural responses measured during the Attention Bias Tasks in the experimental conditions in relation with their respective measures (covariates) in the reference conditions.

(a) Response variables (experimental conditions)	Explanatory variables	
*In the Attention Bias Task*	Covariate (reference)	Housing (positive–negative)
Latency to look at the threat	ß = − 0.063 (0.299), p = 0.820	**p < 0.001**
Latency to eat	ß = 0.317 (0.116), **p = 0.003**	p = 0.950
Time spent looking at the threat	ß = 0.149 (0.202), p = 0.430	p = 0.150
Time spent eating	ß = 0.216 (0.114), **p = 0.043**	p = 0.390
Relative positive attention	ß = 0.109 (0.104), p = 0.260	p = 0.560
Time spent in locomotion	ß = 0.417 (0.173), **p = 0.010**	**p = 0.009**
Time spent in contact with walls/floors	ß = − 0.026 (0.170), p = 0.870	p = 0.320

(b) Response variables	Explanatory variables	
*In the Attention Bias Task*	Covariate (reference)	
Time spent with head up in positive housing	ß = − 0.396 (0.172), **p = 0.011**	
Time spent with head up in negat ive housing	ß = 0.224 (0.195), p = 0.210	

(a) Presents the parameters of the equation lines for both levels of housing, when no significant interaction between the covariate and housing were found. (b) Presents the parameters of the equation line for each level of housing, when an interaction between the covariate and housing was found.

Significant values are in bold.

### Exploratory analyses: personality and housing interactions on heifers’ responses to the judgement and attention bias tests in the experimental conditions

Transverse analyses were used to tentatively explore whether the identified personality traits, *specifically*, may influence individual responses to the cognitive bias tests when heifers were housed under the experimental housing conditions. Regarding the JBT results, there was an interaction between housing (positive versus negative) and Fearfulness (p = 0.007) on *Average latency to reach the ambiguous cues*. Non-fearful heifers were faster to reach the ambiguous cues than fearful heifers in the positive conditions only (Non-fearful: 34% ± 8.9, Fearful: 66% ± 7.0, p = 0.014). There was also an interaction between Activity and Sociability (p = 0.004). Inactive social heifers were slower to reach the ambiguous cues than inactive non-social heifers in the experimental conditions (Inactive social: 72% ± 8.0, Inactiv e non-social: 52% ± 8.8, p = 0.038). Other relations were not significant (for more detail, see the Supplementary Table S1).

Regarding the ABT results, neither housing nor personality significantly influenced heifers’ *Latency to eat* or *Time spent eating*. There was no evidence that housing significantly influenced *Relative positive attention* either (positive: 78% ± 5.6, negative: 78% ± 8.1, p = 0.620), but *Relative positive attention* was lower for fearful heifers than non-fearful heifers (fearful: 68% ± 7.6, non-fearful: 86% ± 5.0, p = 0.002) and for social heifers than non-social heifers (social: 75% ± 7.6, non-social: 80% ± 5.2, p = 0.033). There was a significant interaction between housing and Activity on *Latency to look at the threat* (p < 0.001). Inactive heifers under the positive conditions looked at the threat sooner than inactive heifers under the negative conditions (positive: 1% ± 0.4, negative: 13% ± 5.5, p = 0.003). Furthermore, under the negative housing, active heifers looked at the threat sooner than inactive heifers (active: 6% ± 2.4, p = 0.014). There was also a significant interaction of housing and Sociability on *Latency to look at the threat* (p = 0.013 ). Non-social heifers under the positive conditions looked at the threat sooner than non-social heifers under the negative conditions (positive: 2% ± 0.4, negative: 10% ± 4.6, p = 0.003). Furthermore, under the negative housing, social heifers looked at the threat sooner than non-social heifers (social: 6% ± 2.7, p = 0.012). Similarly, there was a significant interaction between housing and Activity, as well as Fearfulness and Sociability, on *Time spent looking at the threat* (p = 0.035 and p = 0.011, respectively), but subsequent pairwise comparisons did not reveal any significant differences in responses after Bonferroni correction. Housing and personality effects on *Time spent with head up* also appeared. Heifers in the positive conditions spent more time with the head up than heifers in the negative conditions (positive: 20% ± 5.2, negative: 11% ± 3.3, p = 0.005). Regardless of the housing conditions, fearful and social heifers spent more time with the head up than non-fearful and non-social heifers, respectively (fearful: 22% ± 5.9, non-fearful: 11% ± 2.6, p = 0.003; social: 18% ± 5.5, non-social: 13% ± 3.3, p = 0.003). Other relations were not significant (for more detail, see the Supplementary Table S1 online).

### Relationships between heifers’ responses to the judgement and attention biases tests

Under the reference conditions, there was no evidence of significant correlations between any of the behavioural responses obtained in the ABT and the average latency to reach the ambiguous cues in the JBT (p-values between 0.186–0.789). Similarly, there was no evidence of significant correlations between judgement and attention bias responses, after correction for a housing effect (p-values between 0.409–0.906).

## Discussion

The objective of this study was three-fold. First, we investigated the effects of cattle personality on judgement and attention processes while heifers were kept in similar housing conditions to investigate personality-dependent cognitive biases . Heifers were initially housed under reference conditions in an attempt to standardise their background affective states. Second, we investigated the effects of contrasted housing conditions on cattle responses to the JBT and to the ABT by using a longitudinal approach to control for individual differences. Modifications of the housing conditions were used as a procedure to elicit changes in heifers’ affective valence. Third, we examined whether individual variations in the responses to the JBT and to the ABT were consistent across putative affectively-contrasted housing conditions using two complementary approaches (i.e. an untargeted approach using heifers’ response to the JBT/ABT in the reference conditions as a covariate, and a targeted approach focusing on the identified personality traits).

This study supports the idea that cattle personality is multi-dimensional^31,35–38^. We have identified at least three personality traits. RC1 may reflect *Activity/Exploration*. This result is in line with similar studies conducted in cattle^36,39,40^, although one study with a very low number of calves suggested two separate constructs for “Activity” and “Exploration”^37^. RC2 may reflect *Fearfulness* since behaviours reflecting interactions with the NO were strongly correlated on this axis^35,37,41^*.* RC3 may reflect *Sociability*^31^ since it loaded high on latency to reach pen mates and heifers are considered social when they look for conspecifics’ proximity^42^. For ease of reading, the three RCs are hereafter simply referred to as Activity, Fearfulness and Sociability, with a capital.

This study investigated the effect of personality on the perception of ambiguity within the context of a JBT. In our study, inactive fearful heifers were more pessimistic than inactive non-fearful heifers in the reference conditions. Other studies already noted the influence of Activity and Fearfulness on animal judgement biases. Pigs classified as active personality-wise, for instance, were consistently less pessimistic than inactive ones regardless of their housing conditions^33^, and fearful calves consistently showed more pessimistic responses over time than non-fearful individuals^31^. These results may be due to affective differences between animals’ perception of the task and its settings, depending on their personality. Because fearful individuals are prone to neophobia^43^, they may have perceived the exposure to the ambiguous—and intrinsically novel— cues more negatively than non-fearful individuals. Alternatively , the set-up of the JBT itself may have triggered personality variations in pessimism, in an affect-independent manner. Regardless of their affective states, active individuals may be more likely to engage in any kind of locomotor response, e.g. reach the ambiguous cues, than inactive ones. This hypothesis is in agreement with previous research showing that personality, in particular coping style, predicts decision style—which reflects individual predispositions for decisions involving risk/reward trade-offs^44^. Moreover, our study demonstrates that personality profile predicts animal responses to the ambiguous cues better than a unique personality trait does. In our conditions, predispositions to Inactivity and to Fearfulness exerted a synergistic effect on heifers' likelihood to reach the ambiguous cues. To better understand the role of individual differences in judgement processes, we therefore encourage researchers to characterise animal individuality based on personality profile rather than a single personality trait.

This study also investigated the influence of personality on the perception of threat in the context of a newly developed ABT. First, Activity did not significantly influence cattle threat-directed nor fee d-directed behaviours, therefore suggesting that Activity does not alter heifers’ affective perception of threat. This theory is consistent with the idea that Activity is independent from an emotionality dimension^38,45^ and is also in line with Luo and colleagues^22^ who found no significant effect of coping style on *Time spent looking at the threat* or *Time spent eating* in pigs after the threat exposure—although they reported that proactive pigs looked at the threat more frequently than reactive pigs in enriched conditions. As expected, Activity influenced heifers’ locomotor behaviours—active heifers walked more than inactive ones during the ABT. This finding further supports the validity of Activity as a personality trait in cattle, because heifers displayed consistent locomotor behaviours across contexts. Unlike Activity, both Fearfulness and Sociability influenced attention bias in the reference conditions. In humans and farm animals, certain underlying traits, like trait anxiety, have also been associated with sustained attention to threat^14,15,46^ and with longer latencies to engage with positive stimuli^47^. In our conditions, fearful heifers were more biased towards the threat than the bucket (i.e. the positive cue) compared with non-fearful heifers. We suggest that fearful heifers may have experienced the exposure to the dog model more negatively than non-fearful heifers. Considering that heifers in our study had no previous experience with the dog model during the first ABT, we speculate that fearful heifers were more scared of the dog model or more anxious about the threat after the dog model was covered. Moreover, fearful heifers walked more during the ABT compared with non-fearful heifers. This finding is in accordance with studies demonstrating that drug-induced anxiety increases locomotion in hens^47^ and beef cattle^15^ during ABTs. Our results must, nonetheless, be interpreted with caution, since we did not validate beforehand that our dog model was truly perceived as threatening for heifers. Our ABT was adapted from Lee et al.^15^, who validated their task as a reliable tool to assess beef cattle anxiety. However, Lee et al.^15^ were authorised to use a live dog, a procedure against the safety hazard policy of our experimental farm. We also found that social heifers looked faster at the threat than non-social heifers, which may suggest that social heifers were in worse affective states than non-social heifers. This presupposed difference in affective states could be explained by the fact that social heifers may have suffered more than non-social heifers from being separated from their companions during the ABT. In addition, we found that social heifers spent more time in contact with the floor/walls of the arena than non-social heifers. We speculate that this behaviour may reflect social heifers’ heightened motivation to find an exit from the arena in order to reunite with their pen mates, since heifers classified as social in our study were—by definition—more willing to stay in proximity to their conspecifics than non-social heifers. From an evolutionary perspective, and in line with this idea, we hypothesize that social heifers may be more susceptible than non-social heifers to anti-predation grouping, an adaptive strategy used by ungulates to dilute predator risks^48^. This presumed susceptibility to grouping might, therefore, have mediated social heifers’ motivation to escape the arena in response to our predator-like dog model. Finally, similar to JBT findings, we found an interaction effect of personality traits on responses to the ABT, with non-fearful and non-social heifers spending more time eating than heifers of other personality profiles. This finding, once more, highlights the need to characterize individuality among heifers using personality profiles rather than personality traits.

Overall, this study confirms that stable traits in cattle are associated with differences in behaviours during the ABT, as demonstrated by Lee et al.^15^, who showed that beef cattle temperament index (measured from flight speed and crush score) was positively associated with the number of zones crossed and the attention towards the threat. The exact nature of the personality traits underlying this temperament index remains, however, unclear. Lee et al.^15^ suggested that their index reflected individual general agitation, a theory partially supported by our findings showing that Activity influences cattle locomotor behaviour in the ABT. However, since differences in Activity do not explain variation in threat-related behaviours, we suggest that other personality traits, like Sociability, may underlie this temperament index.

Altogether, these results further demonstrate that JBT and ABT paradigms are not purely state-sensitive but are also trait-sensitive. These results may reflect variations in heifers’ background affective states, which could be either due to personality-based differences in individual perception of the reference conditions or due to a failure to standardize background affective states within 9 weeks. Alternatively, our findings may reflect personality-based differences in heifers’ perception of the tasks’ set-up itself (e.g. with regard to the type of response, type of cue, level of isolation, etc.). This idea questions the generalizability of our cognitive bias tasks across individual of various personality and highlights the need to control for individual variations when assessing cognitive biases in our study.

This study also investigated the sole effect of housing on judgement processes, while controlling for individual differences. Surprisingly, changes in housing did not impact heifers' pessimism. One explanation could be that housing did not elicit the expected shift in heifers'  affective states. Background affective states are thought to result from the accumulation of positive and negative experiences^49^, but our housing changes may have been too infrequent (i.e. once a week for an entire week) and predictable (i.e. every Friday afternoon) to truly impact heifers’ opportunity to experience positive or negative events. Alternatively, background affective states may not emerge from a general accumulation of positive and negative experiences, as initially hypothesised, but they may arise—more specifically—from an accumulation of mismatches^50,51^. More research is required to understand the aetiology of background affective states^50^. Another explanation could be that heifers were affected by the changes, but in the short-term only. In our conditions, heifers might have had the ability to habituate to the housing changes within days, while our experiment was designed to detect the long-lasting consequences of housing changes by exposing heifers to the JBT a week after the last housing modifications. Cows may, therefore, be more resilient to successive changes than initially anticipated. Another explanation is that the JBT itself failed to detect the affective difference between heifers housed in contrasted housing conditions. This lack of treatment detection may be due to an impaired sensitivity of our own JBT set-up. We used a Go/NoGo task based on a spatial discrimination among a female population—while a recent systematic review revealed that JBTs yield larger treatment-induced judgement biases when using Go/Go tasks based on auditory or tactile cues in males^20^. Therefore, we cannot exclude the possibility that our housing conditions effectively influenced heifer affective states but that our JBT set-up was not sensitive enough to detect shifts in judgement bias. Our results are in line with Crump and colleagues^52^ who also failed to detect a shift in cows’ pessimism given access to pasture, while using a similar Go/NoGo spatial JBT. Therefore, we encourage researchers to develop alternatives of our JBT set-up (e.g. auditory Go/Go tasks as previously suggested^53^) that would be more sensitive to affective shifts when investigating the effects of common farm practices on cow affective states. Once more, this study highlights the necessity for researchers to combine indicators of various nature (cognitive, behavioural and physiological) to assess animal affective states reliably. During our experiment, samples from various biofluids were collected on a weekly basis during both reference and experimental conditions. Samples were also collected while the heifers were exposed to an acute-stress test at the end of both reference and experimental conditions. Results from these physiological markers (in prep.) will allow us to draw more solid conclusions with regard to potential treatment-induced affective states in heifers. In particular, heifers’ physiological responses to the acute-stress tests will help us identify whether housing effectively influenced individuals’ ability to cope with stressors, since long-term negative affective states are often associated with physiological dysregulations^54^.

As for JBT, this study investigated the sole effects of housing on behavioural responses to the ABT, while controlling for individual differences. Contrary to expectations, heifers in the negative conditions looked at the threat later and walked less than heifers in the positive and in the reference conditions. Furthermore, although non-significant, heifers in the negative conditions spent on average less time with the head up than heifers in the reference conditions. Although unexpected, these results are in line with another study conducted in sheep where chronically stressed individuals exhibited reduced vigilance towards a live predator threat^55^. As hypothesised by the authors of the aforementioned study, these findings are consistent with a phenomenon known as attentional avoidance, where attention is allocated away from the threat location. Similarly, Bethell and colleagues^56^ reported that rhesus macaques avoided looking at threatening faces of conspecifics following an acute-stress procedure. Interestingly, attentional avoidance effects—as opposed to facilitated attention to threat—have repeatedly been reported in anxious humans when threatening stimuli are presented for long (superior to 20 s) but not short durations^57^. Therefore, considering the duration of our trials, our ABT was potentially more likely to detect anxiety-driven differences in attentional avoidance strategies rather than differences in threat detection. Overall, our results could, therefore, suggest that heifers in the negative housing conditions became chronically stressed. This theory, yet, remains to be verified using validated indicators of chronic stress, such as heart rate variability indices^58^. Alternatively, we cannot rule out the idea that these results could also indicate that heifers in the negative conditions learnt to cope better with challenges due to their repeated exposure to stressors during the experimental periods, or that heifers housed in the negative conditions were in relatively better affective states than heifers housed in the positive conditions. Heifers in the negative conditions may have been temporarily relieved to exit their home pens and became momentarily less scared/anxious during the ABT. This assumption is strengthened by the fact that heifers in the negative conditions spent on average less *Time with the head up,* which is a measure of vigilance^59^, than heifers in the reference and positive conditions—although statistical differences between housing conditions were not significant. Nonetheless, this idea is speculative and remains to be further validated—by comparing, for instance, heifers’ home pen behaviours in the different housing conditions. Besides, the use of attention bias—unlike judgement bias—as a valid indicator of positive affective shift remains to be proven^60^. Finally, heifers in both negative and positive housing conditions spent more time eating and shifted their attention more towards the positive cue during the second ABT compared to the first. Overall, these results may indicate that heifers habituated to the task and became either less scared of the dog model or remembered that the familiar bucket was also filled with concentrates during this task. This theory, however, contrasts with a previous study conducted in rhesus macaques, where a week interval between testing seemed sufficient to supress the effect of repeated testing on animal responses to the ABT^61^.

Overall, there is little evidence that our housing conditions influenced dairy heifers’ cognitive biases when controlling for inter-individual variation. While this finding may suggest that our housing conditions did not substantially influence heifers’ affective states, this lack of statistical support may also reflect methodological limitations of our study. In particular, we question the repeatability of our cognitive bias tasks: we cannot exclude the idea that, in the reference conditions, heifers’ responses to the tests influenced individual affective experience of the tests themselves—which might have, in turn, influenced heifers’ responses to the cognitive bias tests in the experimental conditions. More research is, therefore, required, to assess whether animal prior experience of the cognitive bias tests influences individual responses to subsequent tests.

This study also aimed to investigate whether variations in the responses to the JBT were mediated by underlying traits. Assuming that influences of individual traits on judgement bias are constant over time and across contexts, we explored the predictive value of cattle pessimism in the reference conditions on subsequent pessimism when heifers were under supposedly affectively-contrasted housing conditions. Of note, in this study, we restricted ourselves to the sole investigation of linear relationships between pessimism in the reference conditions and pessimism assessed in the experimental conditions. The regression ANCOVA analysis revealed that pessimistic heifers in the reference conditions remained pessimistic in the experimental conditions. This finding is in line with that of Lecorps et al.^31^ who found that pessimism is constant in calves. This result supports the idea that cattle decision-making under ambiguity is influenced by stable individual characteristics, i.e. personality traits independent from environmental context. This hypothesis is furthermore supported by the fact that inactive social heifers responded in a more pessimistic manner than inactive non-social heifers, irrespective of the experimental housing conditions. Such findings may, once more, reflect personality-based differences in heifers’ affective background or personality-based differences in heifers’ perception of the JBT set-up. A word of caution with regard to these results is, however, due here. Considering the relatively small sample size of our population study, we cannot rule out the possibility that our model failed to detect a significant interaction effect between pessimism in the reference conditions and experimental housing (p = 0.082). In agreement with this idea, pessimism in the reference conditions appeared to more reliably predict pessimism in the negative conditions than in the positive housing conditions. This could suggest that personality-based differences in response to the JBT may be exacerbated during challenging conditions, as found in humans and non-human animals^62,63^. Considering that pessimism may be both affective state- and trait-dependent^64^, the relative lack of consistency between pessimism in the reference conditions and pessimism in the positive housing conditions may indicate a certain variability in the affective states experienced by heifers in the positive conditions compared to heifers in the reference conditions. This theory is strengthened by the interaction effect between Fearfulness and hous ing on heifers’ pessimism—non-fearful heifers being less pessimistic than fearful heifers in the positive, but not in the negative conditions. We suggest that fearful heifers may have experienced the repeated positive changes in their environment less pleasantly than fearful heifers, which resulted in greater affective differences among the two sub-populations. Fearful heifers may have suffered from the weekly changes occurring in their home pen, particularly from the repeated introduction of new enrichment. Similarly, frequent rotation of enrichment objects in parrots was shown to successfully reduce individual fear behaviours—except for the most fearful parrots who displayed even more fear behaviours^65^. Therefore, our findings corroborate the idea that individual differences must be carefully considered when designing animals’ enclosure to improve their welfare.

Finally, we investigated whether variations in the responses to the ABT were mediated by underlying traits. Interestingly, positive linear relationships were found between feeding-directed behaviours in the reference and in the experimental conditions. This result suggests the existence of one (or more) underlying stable traits accounting for inter-individual variations in feed-directed behaviours—a result consistent with Melin et al.^66^, who found that individual differences in dairy cattle explain 84% to 98% of the variation in feeding patterns. Furthermore, our exploratory analyses seem to indicate that Fearfulness may mediate cow feeding-directed behaviours, as suggested elsewhere^67^. More research is, nonetheless, warranted to validate this preliminary finding, since we used a relatively low number of individuals per personality trait and housing conditions in our study. In contrast, there were no linear relationships between threat-directed behaviours observed during the reference and during the experimental conditions. We could hypothesise that threat-directed behaviours are relatively insensitive to trait differences in cattle, but this theory seems unlikely considering recent findings demonstrating a high degree of repeatability (R = 0. 63) in attention to threat over several years in rhesus macaques^61^, and the evidence of stable differences in attention biases among humans according to their trait-anxiety scores^46^. Alternatively, the lack of consistency in threat-directed behaviours may, once more, suggest that heifers experienced the housing con ditions differently depending on their personality. This theory is partially supported by the fact that inactive heifers looked at the threat later than active heifers in the negative conditions. Likewise, non-social heifers looked at the threat later than social heifers in the negative conditions. There are several potential explanations to these preliminary findings. Inactive and non-social heifers may have been more relieved to exit their home pens, and therefore temporarily in better affective states during ABT, than active and social heifers. Conversely, and in congruence with the attentional avoidance theory, inactive and non-social heifers may have been in worse affective states than active and social heifers during ABT. Although mutually exclusive, both theories seem to indicate that inactive and non-social heifers may have experienced the negative conditions more aversively than active and social heifers. However, we cannot rule out the possibility that these personality-based differences in response to the ABT may also be independent from any affective processes. For instance, active heifers may have been less flexible in their responses to the ABT than inactive heifers, as a result of personality-dependant behavioural inflexibility^68^. In line with this idea, proactive pigs were shown to be more optimistic in the JBT independent of their housing conditions^33^. Considering that behavioural flexibility depends on individual personality, we therefore question the generalizability of both cognitive bias tasks across individuals of different personality—since both tasks solely rely on behavioural outcomes. Lastly, no clear relationships between heifers’ responses to ABT and JBT were identified, which is in line with previous finding obtained in sheep^23^. This result could potentially indicate that separate mechanisms underlie the aetiology of attention and judgement biases.

In conclusion, we did not find substantial evidence that housing conditions influenced heifers’ affective states since housing had relatively little effect on heifers’ cognitive biases. Nonetheless, when housing effects on cognitive biases were identified, they appeared to be mediated by heifers’ personality. On the one hand, this result could indicate that heifers’ affective experience of their housing conditions differs according to individual personality. On the other hand, this finding questions the validity of both cognitive bias tasks as repeatable tools for the assessment of affective states, since personality-based differences in response to the JBT and to the ABT may also be affect-unrelated.

## Methods

### Animals and husbandry system

The experiment took place between February 2019 and January 2020. The study was divided into three batches of fifteen weeks each. Each batch was composed of four groups of eleven Friesian Holstein dairy cows. Among the eleven individuals, four animals were focal individuals, while seven animals were companion animals. Focal animals (N = 48) were first parity heifers between the third and seventh lactation month when the batch started. The term “focal group” is used here to refer to a subset of four heifers housed in the same pen. Heifers were pseudo-randomly allocated to their group based on their days in milk (165 d ± 5.5), milk production (25.16 kg ± 0.609) and body weight (606 kg ± 6.0). Companion animals were cows between the second and sixth parity. They were pseudo-randomly allocated to their group based on their parity (3 ± 0.1), milk production (30.39 kg ± 0.663) and body weight (707 kg ± 7.4). All heifers and cows were healthy at the beginning of the experiment (i.e. somatic cell count within normal-range, absence of fever and absence of lameness) and confirmed pregnant. However, one companion animal was removed on week fourteen from the first batch because she was found contagious for para-tuberculosis. Furthermore, one heifer was replaced in the second week of the third batch due to miscarriage.

**Figure 1:**
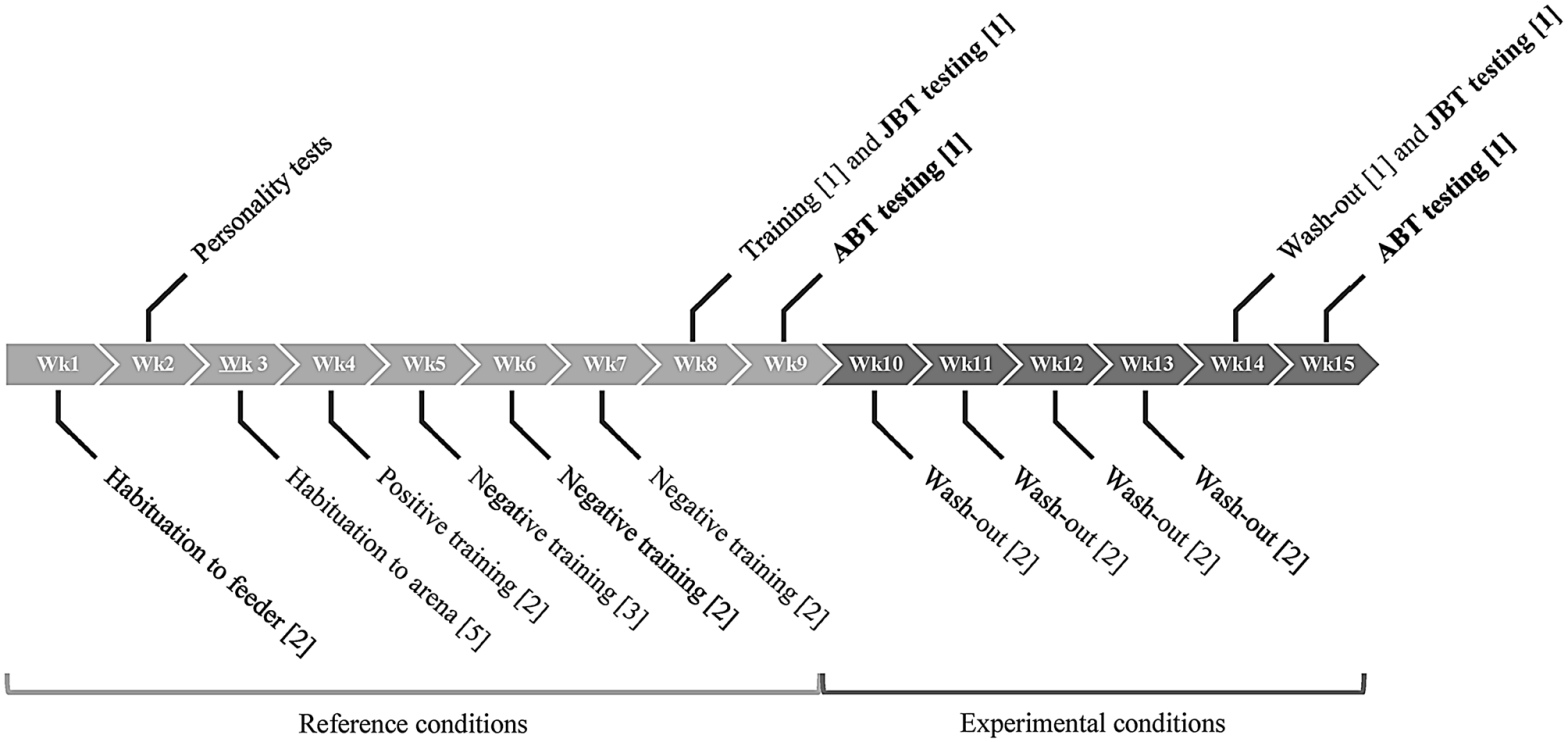
Timeline of the experimental procedures during each batch. Habituation, training and wash-out sessions were part of the Judgement Bias Task (JBT). Numbers in square brackets indicate the number of sessions conducted per heifer per week on weekdays. If necessary, additional sessions were added during the weekends or during the evenings. Testing sessions of the JBT and the Attention Bias Task (ABT) are indicated in bold. Weeks in light grey (week 1–week 9) depict the reference conditions, while weeks in dark grey depict the experimental conditions. Heifers were housed under stable housing conditions during the reference conditions; while they were housed under supposedly weekly-improved or weekly-worsened conditions during the experimental conditions.

All groups were housed in the same barn, but visual and tactile contacts between the groups were prevented via 2 m-high solid partitions. Milking occurred twice a day at around 05:00 h and 15:00 h. Cows received a total mixed ration of maize silage (35% of dry matter), grass silage (30%), concentrates (20%), grinded whole soy (10%), grinded whole wheat (3%) and minerals (2%) at around 7:00 h, which was pushed up again at around 17:00 h. Within a pen, cows had free access to one automatic concentrate dispenser delivering a pre-set daily amount of concentrates based on individual milk production, and they had ad libitum access to one water trough. For each batch, the study was divided into two phases, hereafter referred to as “reference conditions” and “experimental conditions”. At the beginning of our study, heifers were housed in a stable environment in the reference conditions, while they were subsequently housed either under environmental conditions that were assumed to progressively (i.e. every week) worsen, or under environmental conditions that were assumed to progressively improve in the experimental conditions. During both the reference and experimental conditions, heifers were exposed to a series of behavioural tests, including the JBT and the ABT testing (Fig. 1). Heifers’ responses to cognitive bias tests in the reference conditions were used as baseline, while their responses to the tests in the experimental conditions were used as measures of housing-induced affective states.

All experimental procedures were approved by the Central Committee on Animal Experiments (the Hague, the Netherlands), Approval Number AVD4010020174306. All methods involving animals during the study were carried out in accordance with the ‘Wet op de dierproeven’ (law on animal experiments) and ARRIVE guidelines. Methods requiring plant materials were also carried out in accordance with the institutional guidelines and regulations.

### Housing conditions

#### Reference conditions

**Figure 2:**
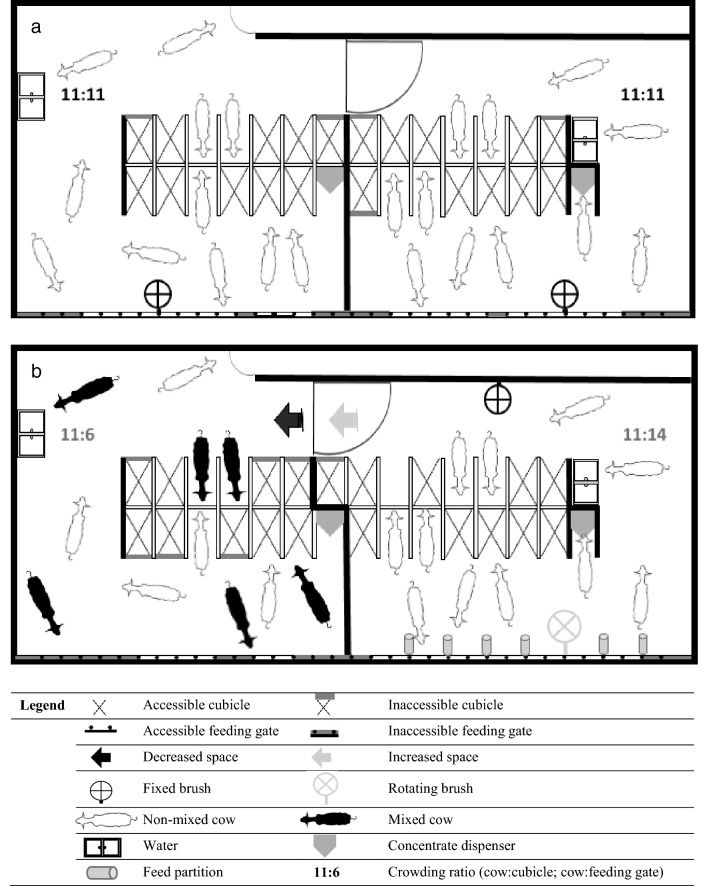
Schematic layout of two adjacent pens. (**a**) Represents a schematic layout of two pens in the reference conditions. (**b**) Represents a schematic layout of two pens at the end of the negative housing conditions (left pen) and at the end of the positive housing conditions (right pen).

For nine weeks, heifers were housed under reference conditions (Fig. 2a). In each pen, cows had access to eleven flexible cubicles with gel mattresses (AgriProm) covered with sawdust, eleven feeding gates, and one simple, fixed brush. Mixing within the groups was prohibited.

#### Experimental conditions

Following the reference conditions, two pens per batch (i.e. six in total) were allocated to weekly-improved housing conditions (hereafter ‘positive housing’), while the other two pens were subjected to weekly-worsened housing conditions (hereafter ‘negative housing’). For a detailed description of the treatments, see Table 6. These weekly changes aimed at inducing positive/negative shifts in heifers’ background affective states, under the assumption that the latter emerge from the accumulation of respectively positive or negative events^49^. To induce a relative positive affective shift, we manipulated the housing conditions based on existing literature in cattle that links specific housing elements with (1) preferential/motivational behaviours, (2) an increase in comfort indicators, or (3) a decrease in behavioural/physiological indicators of stress. Three actions were performed to design the positive housing conditions: under-crowding conditions (extra space^69^, under-crowding^70^), environmental enrichment (provision of brushes^71^, installation of feed partition^72^) and social stability (familiarity among cows^73^). To induce a relative negative affective shift, we manipulated the housing conditions according to the existing literature demonstrating a link between specific housing elements and an increase in cattle physiological or behavioural stress markers. Three actions were performed to design the negative housing conditions: over-crowding^74,75^, barren housing conditions and social instability^76,77^. As a result, the negative housing conditions did not meet the European minimum recommendations—especially in terms of stocking density^78^. In both negative and positive conditions, the weekly housing changes were always performed on Friday afternoons for a period of 1 week. Each experimental week started from Saturdays, i.e. once the weekly treatment had been applied. Successive positive/negative contrasts were thus created, and the risk of heifers habituating to the treatment was therefore minimized. Figure 2b. provides an overview of the housing conditions at the end of the experimental conditions, for both treatments.

**Table 6:** Detailed treatment applied every Friday during the experimental conditions to design the positive and negative housing conditions.

Week	Levers of actions	Positive housing	Negative housing
Week 10	Crowding conditions	Increase space allowance	Decrease space allowance
		Open 1 cubicle and 1 feeding gate	Close 2 cubicles and 2 feeding gates
	Social stability	Add feeding partitions	
Week 11	Enrichment	Add a fixed brush or replace a fixed brush by a rotating one	Remove the fixed brush
	Social stability	Keep stable groups	Mix two companion animals
Week 12	Crowding conditions	Open 1 cubicle and 1 feeding gate	Close 2 cubicles and 2 feeding gates
Week 13	Enrichment	Add a fixed brush or replace a fixed brush by a rotating one	Switch to another homepen
	Social stability	Keep stable groups	Mix two companion animals
Week 14	Crowding conditions	Open 1 cubicle and 1 feeding gate	Close 1 cubicle and 1 feeding gate
	Social stability	Keep stable groups	Mix two companion animals

### Personality tests

On week two, heifers were subjected to three standard personality tests: the Open-Field (OF), Novel-Object (NO) and Runway (RW) tests, in this order. The OF and NO were video recorded (CAMCOLBUL2, Velleman, Belgium), while the RW was live scored. Behaviours were scored using The Observer XT 10 (Noldus Information Technology BV, Wageningen, the Netherlands). For each personality test, the testing order of the experimental groups was pseudo-randomly determined based on pen allocation. Each heifer was consecutively subjected to the OF and NO on the same day. Two days were needed to test all sixteen heifers to the OF and the NO tests. The OF and NO protocols were adapted from those developed for calves by van Reenen et al.^63^. All heifers were subjected to the RW test on the same day. The RW protocol was based on Gibbons et al.^42^. For each test, two experimenters were in charge of handling the cows and scoring the heifers’ behaviours. All behavioural measures are detailed in Table 7.

**Table 7:** Definitions of the behavioural measures recorded or live scored across the three personality tests.

Variable	Definition
**Open-Field and Novel-Object tests**	
In locomotion (% of time)	Movement of front legs or all four legs once one of the two front hooves is off the floor (adapted from van Reenen and colleagues^63^). The locomotion bout stops when both front hooves touch the floor for more than 1 s
In contact with floor and walls (% of time)	Muzzle below heifer’s carpal joint, or head oriented towards the wall with the muzzle in proximity/in contact with the wall
**Novel-Object test**	
Latency to touch the object (s)	Time until the first contact with the object^63^
In contact with the object (% time)	Touching the object with the muzzle, the head or the shoulder
**Runway test**	
Time spent in the 2 m zone (s)	Time spent with both front hooves within 2 m from the gate separating the runway and the waiting area

#### Open-Field test

Groups of three individuals from the same pen were brought into the waiting area. Each group consisted of two heifers and a third parity companion cow. The companion cow was included in the group to prevent each heifer from being isolated in the waiting area while the other heifer was being tested. Heifers were then individually brought into a 7 m × 7 m testing arena, unfamiliar to the animals. Before entering the arena, the focal heifer was positioned inside a 2 m × 1 m starting box, where she remained for 3 min. The door of the testing arena was then manually opened, and the experimenter tapped three times on the heifer’s hips to make her enter. This procedure was applied to ens ure that all heifers entered the arena in a standardised manner. The test started once the heifer crossed the virtual line of the entrance door with two front hooves and it lasted ten minutes.

#### Novel-Object test

Immediately following the OF test, a novel object attached to a rope was quickly lowered in the middle of the arena until it touched the floor. The novel object was then lifted up at 1 m above the floor for 10 min, i.e. for the entire test duration. The novel object was new to the heifers and consisted of two orange cones filled with stones (for weight) and attached together.

#### Runway test

**Figure 3:**
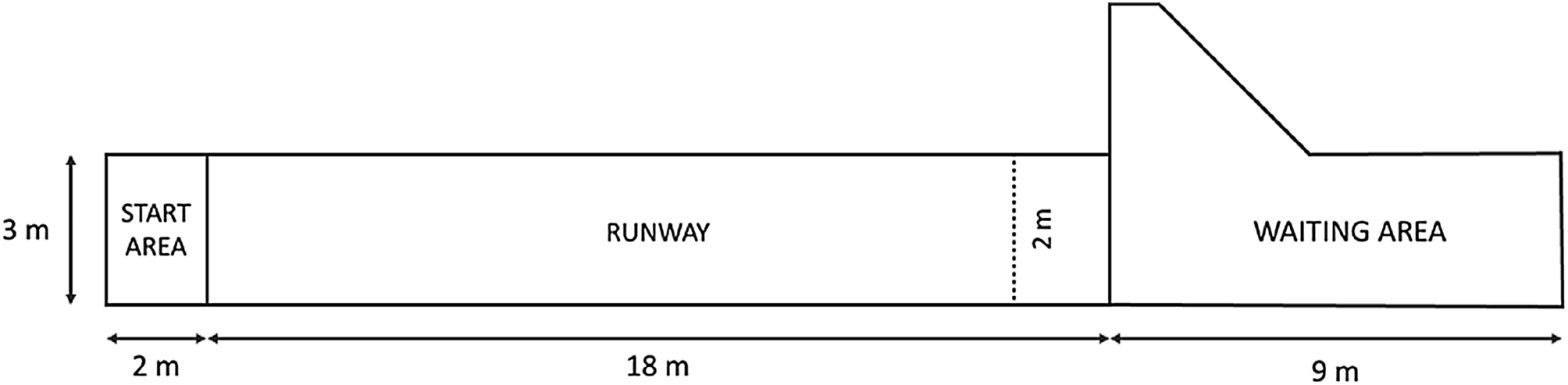
Layout of the runway. During the test, five cows were herded in the waiting area, and the focal heifer was brought by one experimenter to the start area. After 1 min, the heifer was released onto the runway for 5 min. A second experimenter scored the heifer’s time spent within 2 m (dashed line) from the gate separating the runway and the waiting area.

A runway test was conducted in the corridor leading to the milking parlour, (Fig. 3). From each pen, six cows were brought into a waiting area —t he focal group of four heifers and two companion cows of second and third parity. The cows remained in the waiting area for 10 min before the test. Each focal heifer was then tested individually in a random order. The focal heifer was brought by an experimenter into the starting area located 18 m away from the group. A removable bar prevented the heifer to reach the group for 1 min, before being gently removed by the experimenter. The test lasted 5 min and started once the heifer voluntarily crossed the starting line with both front hooves. If the heifer did not cross the starting line within 5 min, the experimenter would encourage the heifer to walk by doing circular forearm movements in the air, without physical contact. At the start of the test, the experimenter slowly withdrew from the runway. During the RW, behaviours were live scored by using a portable computer equipped with The Observer XT 10.

### Judgement bias task

#### Judgement bias apparatus

The Judgement Bias Test (JBT) was carried out in the same arena as the one used for the OF test. It was adapted from previous studies conducted in ruminants^31,53,79^. It consisted of a Go/No-Go task, based on a sp atial cue characterised by an automatic feeder always filled with 150 g of concentrate that could be remotely released. For a detailed description  of the facilities used for the JBT, the reader is referred to Kremer et al.^53^.

#### Judgement bias habituation

The habituation took place in 6 steps. First, a focal group (i.e. four heifers from the same pen) was brought to the waiting area, and pairs of heifers were then habituated to eat from the feeder in a subsection of the waiting area, hereafter called the turning area. Concentrates were released when one heifer was about 50 cm away from the feeder. Once one heifer had eaten from the feeder, she was brought to the exit corridor, to limit competition for the feeder and to allow the second heifer to eat from the feeder. Once the second heifer also ate, the next pair of heifers was brought to the turning area  and the same procedure was applied. Two days later, heifers were once again brought in focal groups of four to the waiting area, but they were individually introduced to the turning area. The focal heifer was released into the exit corridor once she ate three consecutive times from the feeder without startling in response to the concentrate release. Once habituated to the feeder, heifers were familiarised to the testing arena itself two weeks later. Heifers from the same pen were first brought in groups of four inside the testing arena, where three buckets filled with concentrates and one feeder were present in each corner of the arena. Concentrates were remotely released from the feeder when one heifer was 50 cm away from the feeder. The corner attribution was randomly selected. The door of the arena remained open, and the habituation trial stopped once all heifers exited the arena by themselves or after approximatively 5 min. The experi menter then re-filled all buckets and the feeder; and the trial was repeated four to five times in total. On the following day, heifers were brought in pairs inside the testing arena for two consecutive trials of 10 min each. Three buckets and one feeder were located in each corner of the arena. The next day, heifers were introduced alone to the arena for 2 consecutive trials of 5 min each. Heifers still had access to one bucket and one feeder filled with 150 g of concentrates—the positions of which were again pre-randomly selected. Eventually, the feeder was solely positioned in one of the two far corners of the arena (hereafter called ‘positive’ location and abbreviated ‘P'), and the heifer was considered as habituated once she reached the feeder within 3 min for two consecutive habituation trials. Extra habituation sessions were provided until all heifers reached the habituation criterion.

#### Judgement bias positive training

Once habituated, each heifer was subjected to at least two positive training sessions. Each session consisted of three trials of 90 s where the feeder remained in P. The corner attribution was balanced across groups and pens—and remained the same within a pair of heifers for practicality. Before each trial, the heifer was kept for 30 s inside the starting box adjacent to the testing arena. The entrance door was subsequently opened, and the heifer was tapped three consecutive times on her hips to encourage her to enter the arena. If the heifer did not enter, the taps were repeated, and the heifer was eventually physically encouraged if needed. When the heifer’s muzzle reached the 50 cm-radius circle around the feeder, 150 g of concentrate were delivered. If the heifer did not reach the feeder within 90 s, the trial was extended for an additional 30 s. If the heifer still did not reach the feeder, one experimenter entered the arena and gently orientated the heifer towards the feeder while talking to her and petting her on the hips until she reached the feeder and ate from it. Heifers were considered tra ined once they reached the feeder within 30 s for three consecutive times. Additional positive training sessions were provided where necessary.

#### Judgement bias negative training

Following the positive training, heifers were trained to discriminate between two feeder’s locations, either on P or in the opposite corner for at least eight sessions. The opposite corner will hereafter be referred to as ‘N’, which stands for negative location. Heifers were trained to display Go-responses to the feeder to obtain 150 g concentrates when the feeder was located on P. The response was considered correct and deemed a Go when the heifer reached the feeder within 20 s. If she did not reach the feeder within trial duration (90 s), the same procedure as the one used for the positive training session was applied. Alternatively, heifers were trained to display NoGo responses in order to avoid a 6 bar air-puff when the feeder was located on N. This combination of reward/punisher was selected to maximize the sensitivity of our JBT to shifts in heifers’ affective states^53^. The response to N was considered correct and deemed a NoGo if the heifer did not reach the feeder during the whole trial duration, i.e. 90 s. If the heifer reached the feeder during the negative trial, an air puff was released from the bottom of the feeder’s bowl, and the trial ended 10 s later. Each training session was composed of 10 consecutive trials: 6 positive trials, and 4 negative ones. The order of trials was pseudo-randomly determined: the negative training session always started with a positive and a negative trial, and always ended with a positive trial. This was done to ensure heifer’s motivation to participate in the task. From this phase of the JBT, heifers remained 20 s inside the starting box before each trial. Heifer were considered trained if they displayed 10 correct responses during one training session. 

#### Judgement bias testing

On weeks 8 and 14 of the experiment, all heifers were subjected to the JBT. The testing session was composed of 10 consecutive trials, among which 4 positive trials, 3 negative trials and 3 ambiguous trials. The order of trials was pre-determined and the ambiguous trials were interspersed by one positive trial and one negative trial, in this order. Furthermore, the session always started with a positive and a negative trial, and ended with a positive one. All heifers were first exposed to a truly ambiguous cue (A), positioned between P and N. On the sixth and ninth trials, heifers were either exposed to a positive ambiguous cue (Ap) positioned in between A and P; or to a negative ambiguous cue (An) positioned in-between A and N. The order of Ap and An trials was balanced across pairs, groups and treatment. Latencies to reach the cues were video recorded. Animals were exposed to the same sequence of trials on weeks 8 and 14, and were tested exactly in the same order in the two sessions.

#### Judgement bias wash-out

During the experimental conditions, training sessions were maintained. This was made to minimize the risk of heifers remembering their exposure to the ambiguous cues; and to maintain heifers’ routine since the JBT training may provide cognitive enrichment^30^. The same procedures as those used during the training sessions were followed. In total, heifers were exposed to nine wash-out sessions.

### Attention bias task

#### Attention bias apparatus

**Figure 4:**
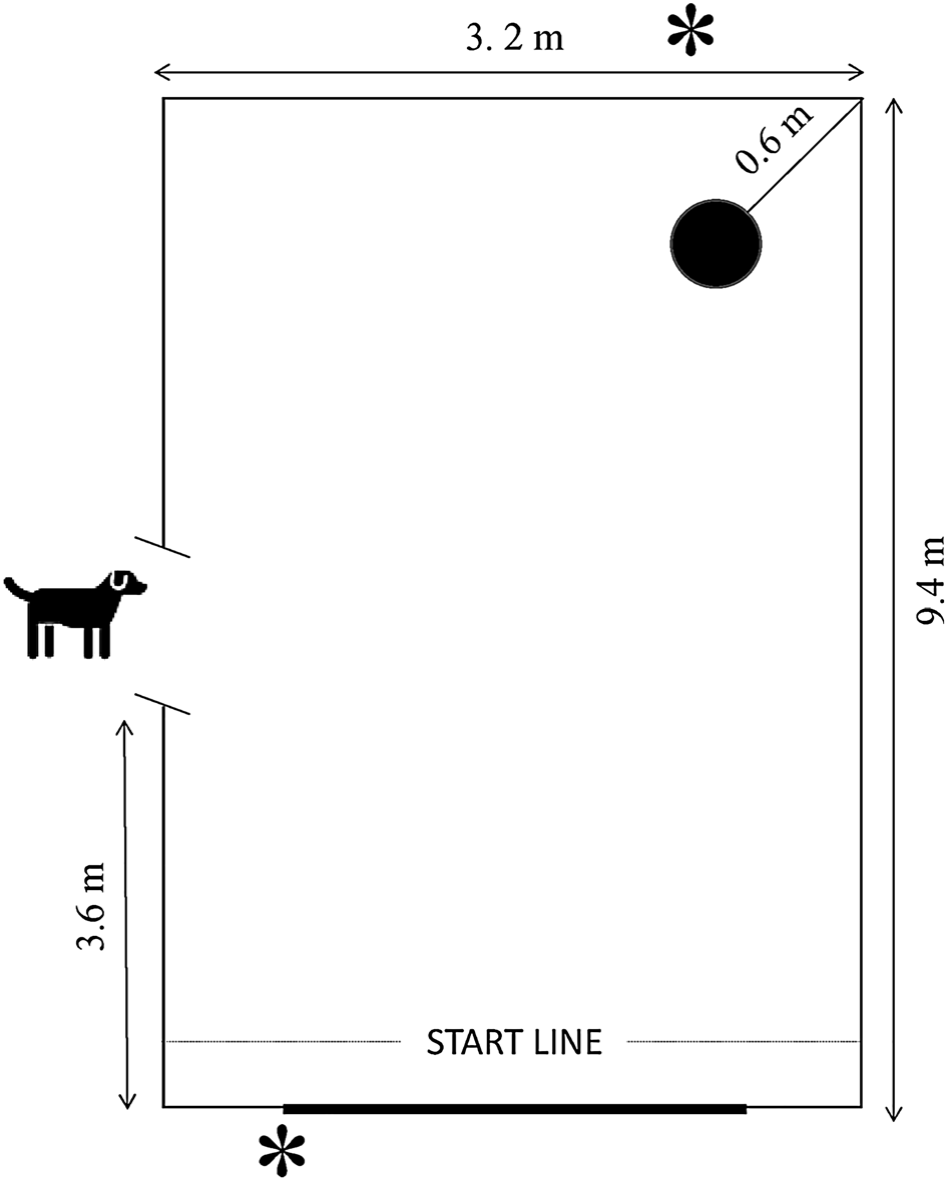
Schematic layout of the attention bias arena. The black circle is the positive cue (i.e. a bucket with 500 g concentrates) and the dog is the threat (dog model). The stars show the locations of the cameras.

The Attention bias task (ABT) was adapted from previous studies conducted in ruminants^14,15^. The arena consisted of a subsection of the milking parlour’s waiting area delimited by a 1.5 to 2.0 m high tarp (Fig. 4). As previously recommended^80^, the positive stimulus and the threat were positioned in a such a way that the heifers could not look at both simultaneously. The positive stimulus consisted of a familiar bucket filled with 500 g of concentrates and was located in the right corner of the arena. The threat consisted of a dog model positioned on the left of the arena, behind a hole (1.0 m × 1.3 m) in the tarp. The dog model was built from a combination of visual, olfactory and auditory cues. In batch 1 and 2, the visual cue consisted of the statue of a blond standing Labrador (73 cm high on a 35 cm elevation). In batch 3, the dog statue was replaced by a sitting brown and white Bulldog (37 cm high on a 61 cm elevation) because the former statue broke prior to the test. The olfactory cue consisted of 2 urine-saturated cotton pads obtained from American Bulldogs (Dierenopvang de Wissel, Leeuwarden, The Netherlands) and positioned underneath the dog statue. Samples were stored at minimum − 18 °C and thawed 24 h before use. The auditory cue was a 5 s recording of a growling dog, played with a Bluetooth speaker located underneath the dog statue.

#### Attention bias test

On weeks 9 and 15 of the experiment, all heifers were subjected to the attention bias test. The order of testing was pseudo-randomised based on the experimental housing conditions and kept identical between the two tests. For practical considerations, heifers from the same focal group were brought together to a waiting arena located approximatively 17 m away from the testing arena. Within a group, heifers’ testing order was randomised. At heifer’s entrance in the arena, the dog statue was visible, and the urine sample’s lid was open. Once the heifer had crossed the start line and made visual contact with the statue, the 5 s auditory cue was played. Ten seconds after the visual contact, the dog model was removed by covering the hole in the tarp and closing the urine sample’s lid. If the heifer did not see the dog statue within 30 s, her attention was drawn to it by playing the auditory cue, and the dog model was hidden 10 s later. The test started once the dog model was removed and heifer’s behaviour was scored during 120 s, from video (Table 8). Faeces were removed between trials.

**Table 8:** Definitions of the behavioural measures recorded during the Attention Bias Tests.

Behaviour	Definition
Attention to the threat	Looks at the closed tarp (next 120 s) with binocular vision—i.e. the head is directed towards the threat
Attention to the bucket	Looks at bucket with binocular vision, in a direct line—i.e. head is directed toward the bucket
Feeding	Places the muzzle within cm from or inside the bucket
Relative positive attention	Time spent looking at the bucket and feeding relative to the total time spent looking at the bucket, feeding and looking at the threat (adapted from^24^)
In locomotion	At least one leg moves
In proximity with walls/floors	Sniffs, touches, licks or chews on the floor or the walls (tarp) of the arena
Head up	Head raised above the withers, when the heifer is not *in proximity with walls/floors*

### Statistical analysis

Datasets and scripts are available in Supplementary Dataset 1 and in Supplementary Script 1, respectively. All statistical analyses were performed using R 4.0.5.

#### Identification of dairy heifers' personality traits

A Principal Component Analysis was used to identify the dimensions of personality among heifers (N = 47). The substitute heifer was excluded from the analysis. PCA analysis followed researchers’ recommendations^81^. In total, six measures were included within the PCA: the proportion of time spent in contact with the object, the latency to touch the object, the proportion of time spent in contact with the walls during the OF and the NO tests, the proportion of time spent in locomotion during the OF and the NO tests, the number of locomotion bouts during the OF and the NO tests, and the proportion of time spent less than 2 m away from the group during the RW test. As recommended, the number of behavioural measures included within the PCA was minimised to ensure an [animals: parameters] ratio superior to 5^81^. The selected behaviours were reduced to behaviours that were not highly correlated with each other (r > 0.7), and behaviours with small in-between animal variability were also disregarded. Latencies were expressed as proportions of total test duration. Latencies and count data were log-transformed (log(y + 1)) or square transformed; and proportion of times were logit transformed (log(y/(1 − y)) using y = 0.1 × minimum(proportion of time) when y = 0^63^ to achieve approximate normality^37^. PCA was performed on the correlation matrix, and the first three factors were included and subjected to varimax rotation. The number of rotated components (RC) was selected based on the number of components explaini ng more than 75% of the total variance. Loadings rated ‘excellent’ (|value| > 0.71^82^) were considered for further interpretation. All communalities were higher than 0.7. Heifers’ scores on the three main components were extracted from the PCA. For each component, heifers were thereafter classified into two classes, based on the component’s median score (RC1: − 0.08, RC2: 0.23, RC3: 0.26).

#### Personality effect on heifers’ responses to judgement and attention bias tests under the reference conditions

The effect of personality traits on heifers’ responses to the cognitive bias tests was assessed in the reference conditions only, i.e. when differences in housing-induced affective states between heifers were minimal. Response variables were expressed as proportions of trial duration (i.e. 90 s for the JBT and 120 s for the ABT) or as proportions of total time spent paying attention to one cue or the other (i.e. *Relative positive attention*). Analyses were conducted using generalized linear mixed models (GLMMs). Analyses were performed by penalized quasi-likelihood^83^ employing routine glmmPQL from the MASS library. The GLMMs comprised of a logit link and a binomial variance function with an extra multiplicative overdispersion parameter. Fixed effects on the logit scale included batch, each personality trait (expressed as two-levels categorical variables), as well as two-way and three-way interactions between personality traits. Random effects included group^84^. Wald tests were performed to assess the main fixed effects in all GLMM analyses. Pairwise comparisons were based on a Fisher’s LSD procedure with Bonferroni correction. Analyses of personality effects on heifers’ responses to the judgement and attention bias tests were conducted on 42 (i.e. trained heifers) and 43 heifers, respectively. Two heifers were excluded from the ABT analyses because they saw the experimenters behind the curtains, and three additional heifers were excluded due to a technical failure of the Bluetooth speaker.

#### Housing effect on heifers’ responses to judgement and attention bias tests

The effect of housing on heifers’ responses to the cognitive bias tests in the reference and in the experimental conditions was investigated in a longitudinal fashion to control for inter-individual variation. Response variables were thus longitudinal data defined as heifers’ behavioural responses to the cognitive bias tests *both* in the reference and in the experimental conditions. Again, analyses were conducted using GLMMs which comprised a logit link and a binomial variance function. Fixed effects included batch and housing (reference, positive, negative) while random effects included heifer nested in group^84^ to account for any source of individual variation, including personality. Analyses of housing effects on heifers’ responses to the JBT and to the ABT were conducted on 41 and 38 heifers, respectively. One trained heifer was removed from the JBT analyses because she suddenly stopped reaching the feeder a week preceding the second testing. Five heifers were removed from ABT analyses because of technical issues.

#### Relationships between cognitive bias responses in the reference and in the experimental conditions

Relationships were investigated using analyses of covariance. Consistency was assessed by investigating the effects of behavioural response *i* to the JBT and to the ABT in the reference conditions on behavioural response *i* to the JBT and to the ABT in the experimental conditions. All responses to the cognitive bias tests were expressed in proportions. For each GLMM, fixed effects included the logit-transformed behavioural response under the reference conditions, housing (positive, negative) and their interaction. The random effects included group. Analyses of responses consistency to the JBT and to the ABT were conducted on 41 and 38 heifers, respectively.

#### Exploratory analyses: personality and housing interactions on heifers’ responses to the judgement and attention bias tests in the experimental conditions

The examination of potential interaction effects between personality and housing on heifers’ responses to the judgement and attention bias tests in the experimental conditions was purely explorative, and not part of the original experimental design. Hence, heifers were not allocated to the positive or to the negative housing conditions based on their personality traits. Consequently, the analyses described are preliminary and the results derived from these analyses should be considered as such. Models were built following a step-by-step approach. The analyses initially included the fixed effects for each personality trait, housing (positive, negative) and all possible two-way and three-way interactions—except for Activity:Fearfulness:Sociability and Treatment:Activity:Sociability due to singularity and convergence issues. From this model skeleton, the selection process of the final models was carried out as follow: (1) removal of three-way interactions with p-values higher than 0.10, (2) removal of two-way interactions between two personality traits with p-values higher than 0.10 and (3) removal of two-way interactions between personality traits and housing with p-values higher than 0.10. Analyses of personality and housing interactions on heifers’ responses to the JBT and to the ABT were conducted on 41 and 38 heifers, respectively.

#### Relationships between heifers’ responses to the judgement and the attention bias tests

Relationships between heifers’ *Average Latency to reach the ambiguous cues* and each behavioural response obtained in the ABT were examined in the reference and in the experimental conditions separately, using Spearman’s rank correlations. In the reference conditions, tests were performed on the raw data expressed as proportions. In the experimental conditions, tests were performed on the residuals extracted from the GLMM analyses modelling the sole effect of housing (positive, negative) on heifers’ responses to the judgement and attention bias tests. In total, 38 and 32 heifers were included for the analyses in the reference and in the experimental conditions, respectively.

## Supplementary Information

Supplementary Information 1.

Supplementary Information 2.

Supplementary Tables.

## Data Availability

All data is available on request and in Supplementary Dataset 1.
