# Supplementary material for: Housing and personality effects on judgement and attention biases in dairy cows
Source: Sci Rep. 2021 Nov 26;11:22984. doi: 10.1038/s41598-021-01843-w (PMC8626508; doi:10.1038/s41598-021-01843-w)
Supplement: Supplementary file 2 — Supplementary Information 2. [file 41598_2021_1843_MOESM2_ESM.docx]

**Housing and personality effects on judgement and attention biases in dairy cows**

Louise Kremer^1,2*^, Jacinta D. Bus^1^, Laura E. Webb^1^, Eddie A. M. Bokkers^1^, Bas Engel^3^, Jozef T.N. van der Werf^2^, Sabine K. Schnabel^3^, Cornelis G. van Reenen^1,2^

^1^ Animal Production Systems Group, Wageningen University & Research, Wageningen, the Netherlands

^2^ Livestock Research, Wageningen University & Research, Wageningen, the Netherlands

^3^ Biometris, Wageningen University & Research, Wageningen, the Netherlands

* Corresponding author. E-mail: [louise.kremer@wur.nl](mailto:louise.kremer@wur.nl)

Scripts below can be copy-pasted and run with R Markdown. Please add a “#” in front of the text (e.g. titles) to run the script.

# SCRIPT FOR PCA

**Dataset**

```{r echo=T, results='hide'}

dataset_sum<-read.table("/Users/louise/Documents/PhD/Papers/JBT-PT/Rebuttal 1/PCA_Manuscript_R1.txt",h=T,row.names=1,check.names=FALSE)

# Rebuttal 1 - check Spearman correlations between NO behaviours

cor.test(dataset_sum$NO_NB_Obj,dataset_sum$Prop_NO_Dur_Obj, method="spearman")

cor.test(dataset_sum$NO_NB_Obj,dataset_sum$NO_Lat_Obj, method="spearman")

cor.test(dataset_sum$RW_Lat_Mid,dataset_sum$Prop_Near_Dur, method="spearman")

# Transform variable to fit approximate normality

shapiro.test(log(dataset_sum$Nb_Loc_All+1))

dataset_sum$Nb_Loc_All<-log(dataset_sum$Nb_Loc_All+1) #ns

sort(dataset_sum$Prop_NO_Dur_Obj)

NO_Prop<-{}

a<-{}

for (i in 1:length(dataset_sum$Prop_NO_Dur_Obj)) {

if (dataset_sum$Prop_NO_Dur_Obj[i]==0) {

a<- log((0.1*0.006)/(1-0.1*0.006))

} else {

a<-log((dataset_sum$Prop_NO_Dur_Obj[i])/(1-dataset_sum$Prop_NO_Dur_Obj[i]))

}

NO_Prop<-c(NO_Prop,a)}

NO_Prop

dataset_sum$Prop_NO_Dur_Obj<-NO_Prop

shapiro.test(dataset_sum$Prop_NO_Dur_Obj) #s

dataset_sum$NO_Lat_Obj<-sqrt(dataset_sum$NO_Lat_Obj)

shapiro.test(dataset_sum$NO_Lat_Obj) #s

# for rebuttal 1 (not applied)

## dataset_sum$RW_Lat_Mid<-sqrt(dataset_sum$RW_Lat_Mid)

## shapiro.test(dataset_sum$RW_Lat_Mid) #s

sort(dataset_sum$Prop_Near_Dur)

Near_Prop<-{}

a<-{}

for (i in 1:length(dataset_sum$Prop_Near_Dur)) {

if (dataset_sum$Prop_Near_Dur[i]==0) {

a<- log((0.1*0.007)/(1-0.1*0.007))

} else {

a<-log((dataset_sum$Prop_Near_Dur[i])/(1-dataset_sum$Prop_Near_Dur[i]))

}

Near_Prop<-c(Near_Prop,a)}

Near_Prop

dataset_sum$Prop_Near_Dur<-Near_Prop

dataset_sum$Prop_Loc_All<-log((dataset_sum$Prop_Loc_All)/(1-dataset_sum$Prop_Loc_All))

shapiro.test(dataset_sum$Prop_Loc_All) # good

dataset_sum$Prop_Walls_All<-log((dataset_sum$Prop_Walls_All)/(1-dataset_sum$Prop_Walls_All))

shapiro.test(dataset_sum$Prop_Walls_All) # good

dataset_sum<-dataset_sum[,c(1,2,3,4,5,6)]

data_matrix_sum = cor(dataset_sum, use = 'complete.obs')

data_matrix_sum

```

**Quality check**

```{r echo=T, results='hide'}

# See intercorrelations variables

round(data_matrix_sum, 2)

# Bartlett's

cortest.bartlett(dataset_sum) # significant good

# KMO<span> (Kaiser-Meyer-Olkin)</span> # should be above 0.6

KMO(data_matrix_sum)

library(REdaS)

bart_spher(data_matrix_sum)

# Determinant

det(data_matrix_sum) # here is 0.16

```

**Analyses**

```{r echo=T, results='hide'}

pc1 = psych::principal(dataset_sum, nfactors = length(dataset_sum), rotate="none",scores=TRUE)

pc1 #3 axis required (SS loadings here are eignevalue)

pc1$scores

apply(pc1$scores,2,var) # scores are rotated

# PCA with 3 axes

pc1 = psych::principal(dataset_sum, nfactors = 3, rotate="none",scores=TRUE) # pc1 with three axe

pc1

pc1$scores # gives the same scores as those with nfactors==length(dataset_sum)

## Final PCA with varimax rotation

pc2 = psych::principal(dataset_sum, nfactors=3, rotate = 'varimax', scores = TRUE) ### based upon correlation matrix

pc2$loadings

pc2$weight

print(pc2$loadings, digits = 8, cutoff = 0.001)

pc2$residual

pc2$fit

pc2$communality

pc2$values # give eigenvalues

dataset <-cbind(dataset_sum, pc2$scores)

summary(dataset$RC1, dataset$RC2)

scores<-dataset[,7:9]

View(scores)

write.csv(dataset_sum, file = "PT_Scores.csv") # extract scores for PTs

# median scores to create binary PTs

median(scores$RC1)

median(scores$RC2)

median(scores$RC3)

````

# SCRIPT FOR COGNITIVE MEASURES IN THE REFERENCE CONDITIONS

```{r}

library(dplyr)

library(emmeans)

library(magrittr)

library(car)

library(MASS)

library(nlme)

library(multcomp)

library(lsmeans)

library(lme4)

library(multcompView)

library(ggplot2)

library(aod)

library(tidyverse)

library(rstatix)

library(broom)

```

**JBT data**

```{r echo=T, results='hide'}

data<-read.table("/Users/louise/Documents/PhD/Papers/JBT-PT/Submission/JBT_Reference_Manuscript_V1.txt",h=T)

str(data)

data$Batch<-as.factor(data$Batch)

data$Cow<-as.factor(data$Cow)

data$Activity<-as.factor(data$Activity)

data$Curiousity<-as.factor(data$Fearfulness)

data$Sociability<-as.factor(data$Sociability)

data$Group<-as.factor(data$Group)

dataset<-subset(data,data$Trained==1) # 927 valid in the reference conditions

data_conv<-subset(dataset,Session=="T0")

nrow(data_conv)

summary(model_1<-glmmPQL(cbind(Prop_Average,100-Prop_Average)~Batch+Activity+Curiousity+Sociability+Activity:Sociability+Activity:Curiousity+Curiousity:Sociability+Activity:Curiousity:Sociability, random=~1|Group, family=quasibinomial, data=data_conv))

summary(model_1<-glmmPQL(cbind(Prop_Average,100-Prop_Average)~Batch+Activity+Curiousity+Sociability+Activity:Sociability+Activity:Curiousity+Curiousity:Sociability, random=~1|Group, family=quasibinomial, data=data_conv))

summary(model_1<-glmmPQL(cbind(Prop_Average,100-Prop_Average)~Batch+Activity+Curiousity+Sociability+Activity:Sociability+Activity:Curiousity+Curiousity:Sociability, random=~1|Group, family=quasibinomial, data=data_conv))

summary(model_f<-glmmPQL(cbind(Prop_Average,100-Prop_Average)~Batch+Activity+Curiousity+Sociability+Activity:Curiousity, random=~1|Group, family=quasibinomial, data=data_conv)) # activity:curiosity interaction

wald.test(vcov(model_f),fixef(model_f), Terms=6) # no sociability effect

wald.test(vcov(model_f),fixef(model_f), Terms=7) # interaction effect

model_obj_em<-emmeans(model_f, ~ Activity:Curiousity)

summary(pairs(model_obj_em),adjust="none") # p values * 6 for manual Bonf. correction

se <- function(x) sqrt(var(x,na.rm=TRUE)/(length(x)-sum(is.na(x)) ))

inact_fear<-subset(data_conv, Activity=="Inact" & Curiousity=="Fear")

mean(inact_fear$Prop_Average)

se(inact_fear$Prop_Average)

inact_nonfear<-subset(data_conv, Activity=="Inact" & Curiousity=="Nonfear")

mean(inact_nonfear$Prop_Average)

se(inact_nonfear$Prop_Average)

```

**ABT data**

```{r}

data<-read.table("/Users/louise/Documents/PhD/Papers/JBT-PT/Rebuttal 1/ABT_Reference_Manuscript_R1.txt",h=T)

se <- function(x) sqrt(var(x,na.rm=TRUE)/(length(x)-sum(is.na(x)) ))

# Latencies are expressed in proportional latencies of total trial duration (120s for ABT)

str(data)

data$Batch<-as.factor(data$Batch)

data$Cow<-as.factor(data$Cow)

data$Activity<-as.factor(data$Activity)

data$Curiousity<-as.factor(data$Fearfulness)

data$Sociability<-as.factor(data$Sociability)

data$Group<-as.factor(data$Group)

dataset<-subset(data,Session=="T0")

nrow(dataset) # 43 correct

dataset_Act<-subset(dataset,Activity=="Act")

cbind(round(apply(dataset_Act[,c(6:15)],2,mean,na.rm=TRUE),0),round(apply(dataset_Act[,c(6:15)],2,se),1)) # all table

dataset_Inact<-subset(dataset,Activity=="Inact")

cbind(round(apply(dataset_Inact[,c(6:15)],2,mean,na.rm=TRUE),0),round(apply(dataset_Inact[,c(6:15)],2,se),1)) # all table

dataset_Fear<-subset(dataset,Curiousity=="Fear")

cbind(round(apply(dataset_Fear[,c(6:15,21)],2,mean,na.rm=TRUE),0),round(apply(dataset_Fear[,c(6:15,21)],2,se),1)) # all table

dataset_Nonfear<-subset(dataset,Curiousity=="Nonfear")

cbind(round(apply(dataset_Nonfear[,c(6:15,21)],2,mean,na.rm=TRUE),0),round(apply(dataset_Nonfear[,c(6:15,21)],2,se),1)) # all table

dataset_Soc<-subset(dataset,Sociability=="Soc")

cbind(round(apply(dataset_Soc[,c(6:15)],2,mean,na.rm=TRUE),0),round(apply(dataset_Soc[,c(6:15)],2,se),1)) # all table

dataset_Nonsoc<-subset(dataset,Sociability=="Nonsoc")

cbind(round(apply(dataset_Nonsoc[,c(6:15)],2,mean,na.rm=TRUE),0),round(apply(dataset_Nonsoc[,c(6:15)],2,se),1)) # all table

### Rebuttal 1 - ABD score

hist(dataset$ABD_prop)

library(lme4)

model_ABD<-lmer(ABD_prop~Batch+Activity+Curiousity+Sociability+(1|Group), data=dataset)

library(ggpubr)

ggqqplot(residuals(model_ABD))

shapiro.test(residuals(model_ABD)) ## excellent

wald.test(vcov(model_ABD),fixef(model_ABD), Terms=4)

wald.test(vcov(model_ABD),fixef(model_ABD), Terms=5) # curiousity effect

wald.test(vcov(model_ABD),fixef(model_ABD), Terms=6)

#### Does it correlate with relative attention?

cor.test(data$ABD_prop,data$Positive_Diff_Att, method="spearman") # strong correlations (ABD excluded from analyses)

### For the latency to see the threat

# Latency to look at the threat

summary(model_1<-glmmPQL(cbind(Atten_Threat_LatinProp,100-Atten_Threat_LatinProp)~Batch+Activity+Curiousity+Sociability+Activity:Sociability+Activity:Curiousity+Curiousity:Sociability+Activity:Curiousity:Sociability, random=~1|Group, family=quasibinomial, data=dataset))

wald.test(vcov(model_1),fixef(model_1), Terms=7) # act:soc

wald.test(vcov(model_1),fixef(model_1), Terms=8)

wald.test(vcov(model_1),fixef(model_1), Terms=9)

wald.test(vcov(model_1),fixef(model_1), Terms=10) # act:cur:soc

summary(model_2<-glmmPQL(cbind(Atten_Threat_LatinProp,100-Atten_Threat_LatinProp)~Batch+Activity+Curiousity+Sociability+Activity:Sociability+Activity:Curiousity+Curiousity:Sociability, random=~1|Group, family=quasibinomial, data=dataset))

wald.test(vcov(model_2),fixef(model_2), Terms=7)

wald.test(vcov(model_2),fixef(model_2), Terms=8)

wald.test(vcov(model_2),fixef(model_2), Terms=9)

## MODEL FINAL

summary(model_3<-glmmPQL(cbind(Atten_Threat_LatinProp,100-Atten_Threat_LatinProp)~Batch+Activity+Curiousity+Sociability, random=~1|Group, family=quasibinomial, data=dataset)) # tendency sociability effect

wald.test(vcov(model_3),fixef(model_3), Terms=4)

wald.test(vcov(model_3),fixef(model_3), Terms=5)

wald.test(vcov(model_3),fixef(model_3), Terms=6)

### For the latency to eat

summary(model_1<-glmmPQL(cbind(Atten_Feeding_LatinProp,100-Atten_Feeding_LatinProp)~Batch+Activity+Curiousity+Sociability+Activity:Sociability+Activity:Curiousity+Curiousity:Sociability+Activity:Curiousity:Sociability, random=~1|Group, family=quasibinomial, data=dataset))

wald.test(vcov(model_1),fixef(model_1), Terms=7)

wald.test(vcov(model_1),fixef(model_1), Terms=8)

wald.test(vcov(model_1),fixef(model_1), Terms=9)

wald.test(vcov(model_1),fixef(model_1), Terms=10)

summary(model_2<-glmmPQL(cbind(Atten_Feeding_LatinProp,100-Atten_Feeding_LatinProp)~Batch+Activity+Curiousity+Sociability+Activity:Sociability+Activity:Curiousity+Curiousity:Sociability, random=~1|Group, family=quasibinomial, data=dataset))

wald.test(vcov(model_2),fixef(model_2), Terms=7)

wald.test(vcov(model_2),fixef(model_2), Terms=8)

wald.test(vcov(model_2),fixef(model_2), Terms=9)

# MODEL FINAL

summary(model_3<-glmmPQL(cbind(Atten_Feeding_LatinProp,100-Atten_Feeding_LatinProp)~Batch+Activity+Curiousity+Sociability, random=~1|Group, family=quasibinomial, data=dataset)) #ns

wald.test(vcov(model_3),fixef(model_3), Terms=4)

wald.test(vcov(model_3),fixef(model_3), Terms=5)

wald.test(vcov(model_3),fixef(model_3), Terms=6)

### For relative attention

summary(model_1<-glmmPQL(cbind(Positive_Diff_Att,100-Positive_Diff_Att)~Batch+Activity+Curiousity+Sociability+Activity:Sociability+Activity:Curiousity+Curiousity:Sociability+Activity:Curiousity:Sociability, random=~1|Group, family=quasibinomial, data=dataset))

wald.test(vcov(model_1),fixef(model_1), Terms=7)

wald.test(vcov(model_1),fixef(model_1), Terms=8)

wald.test(vcov(model_1),fixef(model_1), Terms=9)

wald.test(vcov(model_1),fixef(model_1), Terms=10)

summary(model_2<-glmmPQL(cbind(Positive_Diff_Att,100-Positive_Diff_Att)~Batch+Activity+Curiousity+Sociability+Activity:Sociability+Activity:Curiousity+Curiousity:Sociability, random=~1|Group, family=quasibinomial, data=dataset))

wald.test(vcov(model_2),fixef(model_2), Terms=7)

wald.test(vcov(model_2),fixef(model_2), Terms=8)

wald.test(vcov(model_2),fixef(model_2), Terms=9)

# FINAL MODEL

summary(model_3<-glmmPQL(cbind(Positive_Diff_Att,100-Positive_Diff_Att)~Batch+Activity+Curiousity+Sociability, random=~1|Group, family=quasibinomial, data=dataset))

wald.test(vcov(model_3),fixef(model_3), Terms=4)

wald.test(vcov(model_3),fixef(model_3), Terms=5)

wald.test(vcov(model_3),fixef(model_3), Terms=6)

# curiousity effect

### Proportions of time spent looking at the threat

summary(model_1<-glmmPQL(cbind(Atten_Threat_Prop,100-Atten_Threat_Prop)~Batch+Activity+Curiousity+Sociability+Activity:Sociability+Activity:Curiousity+Curiousity:Sociability+Activity:Curiousity:Sociability, random=~1|Group, family=quasibinomial, data=dataset))

wald.test(vcov(model_1),fixef(model_1), Terms=7)

wald.test(vcov(model_1),fixef(model_1), Terms=8)

wald.test(vcov(model_1),fixef(model_1), Terms=9)

wald.test(vcov(model_1),fixef(model_1), Terms=10)

summary(model_2<-glmmPQL(cbind(Atten_Threat_Prop,100-Atten_Threat_Prop)~Batch+Activity+Curiousity+Sociability+Activity:Sociability+Activity:Curiousity+Curiousity:Sociability, random=~1|Group, family=quasibinomial, data=dataset))

wald.test(vcov(model_2),fixef(model_2), Terms=7)

wald.test(vcov(model_2),fixef(model_2), Terms=8)

wald.test(vcov(model_2),fixef(model_2), Terms=9)

# FINAL MODEL

summary(model_3<-glmmPQL(cbind(Atten_Threat_Prop,100-Atten_Threat_Prop)~Batch+Activity+Curiousity+Sociability, random=~1|Group, family=quasibinomial, data=dataset)) # tendency curiousity effect

wald.test(vcov(model_3),fixef(model_3), Terms=4)

wald.test(vcov(model_3),fixef(model_3), Terms=5)

wald.test(vcov(model_3),fixef(model_3), Terms=6)

### Proportions of time spent eating

summary(model_1<-glmmPQL(cbind(Feeding_Prop,100-Feeding_Prop)~Batch+Activity+Curiousity+Sociability+Activity:Sociability+Activity:Curiousity+Curiousity:Sociability+Activity:Curiousity:Sociability, random=~1|Group, family=quasibinomial, data=dataset))

wald.test(vcov(model_1),fixef(model_1), Terms=7)

wald.test(vcov(model_1),fixef(model_1), Terms=8)

wald.test(vcov(model_1),fixef(model_1), Terms=9)

wald.test(vcov(model_1),fixef(model_1), Terms=10)

summary(model_2<-glmmPQL(cbind(Feeding_Prop,100-Feeding_Prop)~Batch+Activity+Curiousity+Sociability+Activity:Curiousity+Curiousity:Sociability, random=~1|Group, family=quasibinomial, data=dataset))

wald.test(vcov(model_2),fixef(model_2), Terms=7)

wald.test(vcov(model_2),fixef(model_2), Terms=8)

# FINAL MODEL

summary(model_3<-glmmPQL(cbind(Feeding_Prop,100-Feeding_Prop)~Batch+Activity+Curiousity+Sociability+Curiousity:Sociability, random=~1|Group, family=quasibinomial, data=dataset))

wald.test(vcov(model_3),fixef(model_3), Terms=7) # interaction curiousity and sociabiliy

model_obj_em<-emmeans(model_3, ~ Curiousity:Sociability)

summary(pairs(model_obj_em),adjust="none") # p*6 for Bonf. correction

nonfear.nonsoc<-subset(dataset,Curiousity=="Nonfear"&Sociability=="Nonsoc")

mean(nonfear.nonsoc$Feeding_Prop)

se(nonfear.nonsoc$Feeding_Prop)

fear.nonsoc<-subset(dataset,Curiousity=="Fear"&Sociability=="Nonsoc")

mean(fear.nonsoc$Feeding_Prop)

se(fear.nonsoc$Feeding_Prop)

fear.soc<-subset(dataset,Curiousity=="Fear"&Sociability=="Soc")

mean(fear.soc$Feeding_Prop)

se(fear.soc$Feeding_Prop)

nonfear.soc<-subset(dataset,Curiousity=="Nonfear"&Sociability=="Soc")

mean(nonfear.soc$Feeding_Prop)

se(nonfear.soc$Feeding_Prop)

### Proportions of time being vigilant

summary(model_1<-glmmPQL(cbind(Vigi_Prop,100-Vigi_Prop)~Batch+Activity+Curiousity+Sociability+Activity:Sociability+Activity:Curiousity+Curiousity:Sociability+Activity:Curiousity:Sociability, random=~1|Group, family=quasibinomial, data=dataset))

wald.test(vcov(model_1),fixef(model_1), Terms=7)

wald.test(vcov(model_1),fixef(model_1), Terms=8)

wald.test(vcov(model_1),fixef(model_1), Terms=9)

wald.test(vcov(model_1),fixef(model_1), Terms=10)

summary(model_2<-glmmPQL(cbind(Vigi_Prop,100-Vigi_Prop)~Batch+Activity+Curiousity+Sociability+Activity:Sociability+Activity:Curiousity+Curiousity:Sociability, random=~1|Group, family=quasibinomial, data=dataset))

wald.test(vcov(model_2),fixef(model_2), Terms=7)

wald.test(vcov(model_2),fixef(model_2), Terms=8)

wald.test(vcov(model_2),fixef(model_2), Terms=9)

summary(model_3<-glmmPQL(cbind(Vigi_Prop,100-Vigi_Prop)~Batch+Activity+Curiousity+Sociability, random=~1|Group, family=quasibinomial, data=dataset)) # ns here vigilance is linked with both fearfulness and sociability?

wald.test(vcov(model_3),fixef(model_3), Terms=4)

wald.test(vcov(model_3),fixef(model_3), Terms=5)

wald.test(vcov(model_3),fixef(model_3), Terms=6)

boxplot(dataset$Vigi_Prop~dataset$Curiousity)

boxplot(dataset$Vigi_Prop~dataset$Sociability)

### Proportions of time in locomotion

summary(model_1<-glmmPQL(cbind(Loc_Prop,100-Loc_Prop)~Batch+Activity+Curiousity+Sociability+Activity:Sociability+Activity:Curiousity+Curiousity:Sociability+Activity:Curiousity:Sociability, random=~1|Group, family=quasibinomial, data=dataset))

wald.test(vcov(model_1),fixef(model_1), Terms=7)

wald.test(vcov(model_1),fixef(model_1), Terms=8)

wald.test(vcov(model_1),fixef(model_1), Terms=9)

wald.test(vcov(model_1),fixef(model_1), Terms=10)

summary(model_2<-glmmPQL(cbind(Loc_Prop,100-Loc_Prop)~Batch+Activity+Curiousity+Sociability+Activity:Sociability+Activity:Curiousity+Curiousity:Sociability, random=~1|Group, family=quasibinomial, data=dataset))

wald.test(vcov(model_2),fixef(model_2), Terms=7)

wald.test(vcov(model_2),fixef(model_2), Terms=8)

wald.test(vcov(model_2),fixef(model_2), Terms=9)

summary(model_3<-glmmPQL(cbind(Loc_Prop,100-Loc_Prop)~Batch+Activity+Curiousity+Sociability, random=~1|Group, family=quasibinomial, data=dataset)) # activity and curiousity

wald.test(vcov(model_3),fixef(model_3), Terms=4)

wald.test(vcov(model_3),fixef(model_3), Terms=5)

wald.test(vcov(model_3),fixef(model_3), Terms=6)

### Proportions of time in exploration

summary(model_1<-glmmPQL(cbind(Expl_Arena_Prop,100-Expl_Arena_Prop)~Batch+Activity+Curiousity+Sociability+Activity:Sociability+Activity:Curiousity+Curiousity:Sociability+Activity:Curiousity:Sociability, random=~1|Group, family=quasibinomial, data=dataset))

wald.test(vcov(model_1),fixef(model_1), Terms=7)

wald.test(vcov(model_1),fixef(model_1), Terms=8)

wald.test(vcov(model_1),fixef(model_1), Terms=9)

wald.test(vcov(model_1),fixef(model_1), Terms=10)

summary(model_2<-glmmPQL(cbind(Expl_Arena_Prop,100-Expl_Arena_Prop)~Batch+Activity+Curiousity+Sociability+Activity:Sociability+Activity:Curiousity+Curiousity:Sociability, random=~1|Group, family=quasibinomial, data=dataset))

wald.test(vcov(model_2),fixef(model_2), Terms=7)

wald.test(vcov(model_2),fixef(model_2), Terms=8)

wald.test(vcov(model_2),fixef(model_2), Terms=9) # curiousity/sociability dropped but tendency

# FINAL MODEL

summary(model_3<-glmmPQL(cbind(Expl_Arena_Prop,100-Expl_Arena_Prop)~Batch+Activity+Curiousity+Sociability, random=~1|Group, family=quasibinomial, data=dataset))

wald.test(vcov(model_3),fixef(model_3), Terms=4)

wald.test(vcov(model_3),fixef(model_3), Terms=5)

wald.test(vcov(model_3),fixef(model_3), Terms=6)

# Supplementary material 1

# Heifers to be Positive

## For active/inactive

data2_pos_conv_act<-subset(dataset,Treatment=="Positive"&Session=="T0"&Activity=="Act")

data2_pos_conv_inact<-subset(dataset,Treatment=="Positive"&Session=="T0"&Activity=="Inact")

cbind(round(apply(data2_pos_conv_act[,c(13,15,6,8,9,12,10,11)],2,mean,na.rm=TRUE),0),round(apply(data2_pos_conv_act[,c(13,15,6,8,9,12,10,11)],2,se),1))# conv table

cbind(round(apply(data2_pos_conv_inact[,c(13,15,6,8,9,12,10,11)],2,mean,na.rm=TRUE),0),round(apply(data2_pos_conv_inact[,c(13,15,6,8,9,12,10,11)],2,se),1))# conv table

## For fearful/non-fearful

data2_pos_conv_fear<-subset(dataset,Treatment=="Positive"&Session=="T0"&Fearfulness=="Fear")

data2_pos_conv_nonfear<-subset(dataset,Treatment=="Positive"&Session=="T0"&Fearfulness=="Nonfear")

cbind(round(apply(data2_pos_conv_fear[,c(13,15,6,8,9,12,10,11)],2,mean,na.rm=TRUE),0),round(apply(data2_pos_conv_fear[,c(13,15,6,8,9,12,10,11)],2,se),1))# conv table

cbind(round(apply(data2_pos_conv_nonfear[,c(13,15,6,8,9,12,10,11)],2,mean,na.rm=TRUE),0),round(apply(data2_pos_conv_nonfear[,c(13,15,6,8,9,12,10,11)],2,se),1))# conv table

## For social/non-social

data2_pos_conv_soc<-subset(dataset,Treatment=="Positive"&Session=="T0"&Sociability=="Soc")

data2_pos_conv_nonsoc<-subset(dataset,Treatment=="Positive"&Session=="T0"&Sociability=="Nonsoc")

cbind(round(apply(data2_pos_conv_soc[,c(13,15,6,8,9,12,10,11)],2,mean,na.rm=TRUE),0),round(apply(data2_pos_conv_soc[,c(13,15,6,8,9,12,10,11)],2,se),1))# conv table

cbind(round(apply(data2_pos_conv_nonsoc[,c(13,15,6,8,9,12,10,11)],2,mean,na.rm=TRUE),0),round(apply(data2_pos_conv_nonsoc[,c(13,15,6,8,9,12,10,11)],2,se),1))# conv table

# Heifers to be negative

## For active/inactive

data2_neg_conv_act<-subset(dataset,Treatment=="Negative"&Session=="T0"&Activity=="Act")

data2_neg_conv_inact<-subset(dataset,Treatment=="Negative"&Session=="T0"&Activity=="Inact")

cbind(round(apply(data2_neg_conv_act[,c(13,15,6,8,9,12,10,11)],2,mean,na.rm=TRUE),0),round(apply(data2_neg_conv_act[,c(13,15,6,8,9,12,10,11)],2,se),1))# conv table

cbind(round(apply(data2_neg_conv_inact[,c(13,15,6,8,9,12,10,11)],2,mean,na.rm=TRUE),0),round(apply(data2_neg_conv_inact[,c(13,15,6,8,9,12,10,11)],2,se),1))# conv table

## For fearful/non-fearful

data2_neg_conv_fear<-subset(dataset,Treatment=="Negative"&Session=="T0"&Fearfulness=="Fear")

data2_neg_conv_nonfear<-subset(dataset,Treatment=="Negative"&Session=="T0"&Fearfulness=="Nonfear")

cbind(round(apply(data2_neg_conv_fear[,c(13,15,6,8,9,12,10,11)],2,mean,na.rm=TRUE),0),round(apply(data2_neg_conv_fear[,c(13,15,6,8,9,12,10,11)],2,se),1))# conv table

cbind(round(apply(data2_neg_conv_nonfear[,c(13,15,6,8,9,12,10,11)],2,mean,na.rm=TRUE),0),round(apply(data2_neg_conv_nonfear[,c(13,15,6,8,9,12,10,11)],2,se),1))# conv table

## For social/non-social

data2_neg_conv_soc<-subset(dataset,Treatment=="Negative"&Session=="T0"&Sociability=="Soc")

data2_neg_conv_nonsoc<-subset(dataset,Treatment=="Negative"&Session=="T0"&Sociability=="Nonsoc")

cbind(round(apply(data2_neg_conv_soc[,c(13,15,6,8,9,12,10,11)],2,mean,na.rm=TRUE),0),round(apply(data2_neg_conv_soc[,c(13,15,6,8,9,12,10,11)],2,se),1))# conv table

cbind(round(apply(data2_neg_conv_nonsoc[,c(13,15,6,8,9,12,10,11)],2,mean,na.rm=TRUE),0),round(apply(data2_neg_conv_nonsoc[,c(13,15,6,8,9,12,10,11)],2,se),1))# conv table

```

# SCRIPT FOR COGNITIVE MEASURES IN THE EXPERIMENTAL CONDITIONS

```{r echo=T, results='hide'}

library(dplyr)

library(emmeans)

library(magrittr)

library(knitr)

library(car)

library(MASS)

library(nlme)

library(multcomp)

library(lsmeans)

library(lme4)

library(multcompView)

library(ggplot2)

library(aod)

library(tidyverse)

library(rstatix)

library(broom)

```

**JBT data**

Here we consider only one response variable for the JBT - that is the average latency to reach all three cues (expressed in proportion of trial duration)

```{r echo=T, results='hide'}

data<-read.table("/Users/louise/Documents/PhD/Papers/JBT-PT/Submission/JBT_Exp_Manuscript_V1.txt",h=T)

str(data)

data$Batch<-as.factor(data$Batch)

data$Treatment<-as.factor(data$Treatment)

data$Cow<-as.factor(data$Cow)

data$Activity<-as.factor(data$Activity)

data$Curiousity<-as.factor(data$Fearfulness)

data$Sociability<-as.factor(data$Sociability)

data$Group<-as.factor(data$Group)

nrow(data) # should be 41

str(data)

```

**Strategy 1: extract residuals**

```{r echo=T, results='hide'}

summary(mo1<-glmmPQL(cbind(Prop_Average_Exp,100-Prop_Average_Exp)~Batch+Treatment,random=~1|Group, family=quasibinomial, data=data))

summary(mo1)$tT

wald.test(vcov(mo1),fixef(mo1), Terms=1:3)

wald.test(vcov(mo1),fixef(mo1), Terms=4)

res_JBT<-as.vector(residuals(mo1))

data_JBT_res<-cbind(data,res_JBT)

write.csv(data_JBT_res, file = "JBT_Exp_Res.csv")

data$res_JBT<-res_JBT

```

**Strategy 1b: investigate the effect of all three housing conditions (only) on JBT responses**

```{r echo=T, results='hide'}

data2<-read.table("/Users/louise/Documents/PhD/Papers/JBT-PT/Submission/JBT_3Housing_Manuscript_V1.txt",h=T)

str(data2)

data2$Batch<-as.factor(data2$Batch)

data2$Housing<-as.factor(data2$Housing)

data2$Cow<-as.factor(data2$Cow)

data2$Activity<-as.factor(data2$Activity)

data2$Curiousity<-as.factor(data2$Fearfulness)

data2$Sociability<-as.factor(data2$Sociability)

data2$Group<-as.factor(data2$Group)

nrow(data2) # should be 82

str(data2)

summary(mo1<-glmmPQL(cbind(Prop_Average_Exp,100-Prop_Average_Exp)~Batch+Housing,random=~1|Group/Cow, family=quasibinomial, data=data2))

wald.test(vcov(mo1),fixef(mo1), Terms=4:5)

se <- function(x) sqrt(var(x,na.rm=TRUE)/(length(x)-sum(is.na(x)) ))

data2_conv<-subset(data2,Housing=="Reference")

data2_pos<-subset(data2,Housing=="Positive")

data2_neg<-subset(data2,Housing=="Negative")

cbind(round(mean(data2_conv$Prop_Average_Exp,na.rm=TRUE),0),round(se(data2_conv$Prop_Average_Exp),1))

cbind(round(mean(data2_pos$Prop_Average_Exp,na.rm=TRUE),0),round(se(data2_pos$Prop_Average_Exp),1))

cbind(round(mean(data2_neg$Prop_Average_Exp,na.rm=TRUE),0),round(se(data2_neg$Prop_Average_Exp),1))

model_obj_em<-emmeans(mo1, ~ Housing)

summary(pairs(model_obj_em),adjust="none")

# Graph for housing

data2 %>%

group_by(Housing) %>%

ggplot(aes(x=Housing, y=Prop_Average_Exp, fill=Housing)) +

geom_boxplot() +

theme_ipsum() +

theme(

legend.position="right",

plot.title = element_text(size=11)

) +

ggtitle("Average latency to reach the three ambiguous cues according to the housing)") +

xlab("")

```

**Strategy 2: account for individual differences (of all types including potentially PT effect) using responses under the reference conditions as covariates**

```{r echo=T, results='hide'}

# logit function: is it correct?

logit <- function(x) log((x+0.5)/(100-x+0.5))

# Put the covariate on the logit scale

data$Logit_Average_Conv<-logit(data$Prop_Average_Ref)

hist(data$Logit_Average_Conv)

hist(data$Prop_Average_Ref)

summary(mo1<-glmmPQL(cbind(Prop_Average_Exp,100-Prop_Average_Exp)~Batch+Treatment+Logit_Average_Conv+Treatment:Logit_Average_Conv,random=~1|Group, family=quasibinomial, data=data)) #

wald.test(vcov(mo1),fixef(mo1), Terms=6) # ns interaction (0.082)

summary(mo1b<-glmmPQL(cbind(Prop_Average_Exp,100-Prop_Average_Exp)~Batch+Treatment+Treatment:Logit_Average_Conv,random=~1|Group, family=quasibinomial, data=data)) #

summary(mo2<-glmmPQL(cbind(Prop_Average_Exp,100-Prop_Average_Exp)~Batch+Treatment+Logit_Average_Conv,random=~1|Group, family=quasibinomial, data=data))

wald.test(vcov(mo2),fixef(mo2), Terms=4) #

wald.test(vcov(mo2),fixef(mo2), Terms=5) # logit effect

```

### Strategy 2b

```{r echo=T, results='hide'}

# to extract independent Beta

summary(mo2<-glmmPQL(cbind(Prop_Average_Exp,100-Prop_Average_Exp)~Batch+Treatment+Treatment:Logit_Average_Conv,random=~1|Group, family=quasibinomial, data=data)) #

dataset_neg<-subset(data,Treatment=="Negative")

plot(logit(dataset_neg$Prop_Average_Exp)~dataset_neg$Logit_Average_Conv)

```

**Strategy 3: exploratory analyses to investigate the influence of personality traits on the JBT responses**

**Transverse approach**

```{r echo=T, results='hide'}

summary(mo3<-glmmPQL(cbind(Prop_Average_Exp,100-Prop_Average_Exp)~Batch+Treatment+Activity+Curiousity+Sociability+Treatment:Activity+Treatment:Curiousity+Treatment:Sociability+Activity:Curiousity+Activity:Sociability+Curiousity:Sociability+Treatment:Activity:Curiousity+Treatment:Activity:Sociability+Treatment:Curiousity:Sociability+Activity:Curiousity:Sociability,random=~1|Group, family=quasibinomial, data=data))

wald.test(vcov(mo3),fixef(mo3), Terms=17) #

# S1

summary(mo3<-glmmPQL(cbind(Prop_Average_Exp,100-Prop_Average_Exp)~Batch+Treatment+Activity+Curiousity+Sociability+Treatment:Activity+Treatment:Curiousity+Treatment:Sociability+Activity:Curiousity+Activity:Sociability+Curiousity:Sociability+Treatment:Activity:Curiousity+Treatment:Activity:Sociability+Treatment:Curiousity:Sociability,random=~1|Group, family=quasibinomial, data=data))

wald.test(vcov(mo3),fixef(mo3), Terms=14) #

wald.test(vcov(mo3),fixef(mo3), Terms=15) #

wald.test(vcov(mo3),fixef(mo3), Terms=16) #

summary(mo3_1<-glmmPQL(cbind(Prop_Average_Exp,100-Prop_Average_Exp)~Batch+Treatment+Activity+Curiousity+Sociability+Treatment:Activity+Treatment:Curiousity+Treatment:Sociability+Activity:Curiousity+Activity:Sociability+Curiousity:Sociability,random=~1|Group, family=quasibinomial, data=data))

wald.test(vcov(mo3_1),fixef(mo3_1), Terms=11) #

wald.test(vcov(mo3_1),fixef(mo3_1), Terms=12) #s

wald.test(vcov(mo3_1),fixef(mo3_1), Terms=13) #

# S2

summary(mo3_2<-glmmPQL(cbind(Prop_Average_Exp,100-Prop_Average_Exp)~Batch+Treatment+Activity+Curiousity+Sociability+ +Treatment:Activity+Treatment:Curiousity+Treatment:Sociability+Activity:Sociability,random=~1|Group, family=quasibinomial, data=data))

wald.test(vcov(mo3_2),fixef(mo3_2), Terms=8) #

wald.test(vcov(mo3_2),fixef(mo3_2), Terms=9) # s

wald.test(vcov(mo3_2),fixef(mo3_2), Terms=10) #

wald.test(vcov(mo3_2),fixef(mo3_2), Terms=11) # s

# S3

summary(mo3_f<-glmmPQL(cbind(Prop_Average_Exp,100-Prop_Average_Exp)~Batch+Treatment+Activity+Curiousity+Sociability+ +Treatment:Curiousity+Activity:Sociability,random=~1|Group, family=quasibinomial, data=data))

wald.test(vcov(mo3_f),fixef(mo3_f), Terms=1:3)

wald.test(vcov(mo3_f),fixef(mo3_f), Terms=4)

wald.test(vcov(mo3_f),fixef(mo3_f), Terms=5)

wald.test(vcov(mo3_f),fixef(mo3_f), Terms=6)

wald.test(vcov(mo3_f),fixef(mo3_f), Terms=7)

wald.test(vcov(mo3_f),fixef(mo3_f), Terms=8)

wald.test(vcov(mo3_f),fixef(mo3_f), Terms=9)

model_obj_em<-emmeans(mo3_f, ~ Treatment:Curiousity)

summary(pairs(model_obj_em),adjust="none") # 4 contrasts of interest

# Negative,Nonfear - Positive,Nonfear 1.1624 0.578 8 2.012 0.0791

# Negative,Nonfear - Negative,Fear 0.0584 0.569 24 0.103 0.9192

# Positive,Nonfear - Positive,Fear -2.0611 0.634 24 -3.250 0.0034 ## *4

# Negative,Fear - Positive,Fear -0.9570 0.623 8 -1.535 0.1633

model_obj_em<-emmeans(mo3_f, ~ Activity:Sociability)

summary(pairs(model_obj_em),adjust="none") # 6 contrasts of interest

# Act,Nonsoc - Inact,Nonsoc 0.901 0.618 24 1.458 0.1578

# Act,Nonsoc - Act,Soc 0.295 0.561 24 0.525 0.6041

# Act,Nonsoc - Inact,Soc -1.074 0.560 24 -1.920 0.0668

# Inact,Nonsoc - Act,Soc -0.607 0.610 24 -0.994 0.3299

# Inact,Nonsoc - Inact,Soc -1.976 0.662 24 -2.984 0.0064 ## *6

# Act,Soc - Inact,Soc -1.369 0.571 24 -2.397 0.0247

```

**ABT data**

```{r echo=T, results='hide'}

data<-read.table("/Users/louise/Documents/PhD/Papers/JBT-PT/Rebuttal 1/ABT_Exp_Manuscript_R1.txt",h=T)

str(data)

data$Batch<-as.factor(data$Batch)

data$Treatment<-as.factor(data$Treatment)

data$Cow<-as.factor(data$Cow)

data$Activity<-as.factor(data$Activity)

data$Curiousity<-as.factor(data$Fearfulness)

data$Sociability<-as.factor(data$Sociability)

data$Group<-as.factor(data$Group)

nrow(data) # should be 38

str(data)

```

**Strategy 1: extract residuals**

```{r echo=T, results='hide'}

**Transverse approach**

# Transverse approach only

# Rebuttal 1 - ABD score

moABD<-lmer(ABD_Prop_Exp~Batch+Treatment+(1|Group), data=data)

ggqqplot(residuals(moABD)) # good

wald.test(vcov(moABD),fixef(moABD), Terms=4) # 0.67

summary(mo11<-glmmPQL(cbind(Atten_Threat_Prop_Exp,100-Atten_Threat_Prop_Exp)~Batch+Treatment,random=~1|Group, family=quasibinomial, data=data))

res_ABT_Threat_Prop<-as.vector(residuals(mo11))

data_ABT_res<-cbind(data,res_ABT_Threat_Prop)

wald.test(vcov(mo11),fixef(mo11), Terms=4) #

summary(mo13<-glmmPQL(cbind(Feeding_Prop_Exp,100-Feeding_Prop_Exp)~Batch+Treatment,random=~1|Group, family=quasibinomial, data=data)) #ns

wald.test(vcov(mo13),fixef(mo13), Terms=4) #

res_ABT_Feeding_Prop<-as.vector(residuals(mo13))

data_ABT_res<-cbind(data_ABT_res,res_ABT_Feeding_Prop)

summary(mo14<-glmmPQL(cbind(Positive_Diff_Att_Exp,100-Positive_Diff_Att_Exp)~Batch+Treatment,random=~1|Group, family=quasibinomial, data=data)) #ns

wald.test(vcov(mo14),fixef(mo14), Terms=4) #

res_ABT_Pos_Diff_Att<-as.vector(residuals(mo14))

data_ABT_res<-cbind(data_ABT_res,res_ABT_Pos_Diff_Att)

summary(mo15<-glmmPQL(cbind(Atten_Threat_LatinProp_Exp,100-Atten_Threat_LatinProp_Exp)~Batch+Treatment,random=~1|Group, family=quasibinomial, data=data))# diff

wald.test(vcov(mo15),fixef(mo15), Terms=4) #

res_ABT_Threat_Lat<-as.vector(residuals(mo15))

res_1<-res_ABT_Threat_Lat[1:19]

res_2<-c(res_1,NA)

res_3<-res_ABT_Threat_Lat[20:27]

res_4<-c(res_2,res_3,NA)

res_5<-res_ABT_Threat_Lat[28:36]

res_ABT_Threat_Lat_NA<-c(res_4,res_5)

data_ABT_res<-cbind(data_ABT_res,res_ABT_Threat_Lat_NA)

summary(mo17<-glmmPQL(cbind(Atten_Feeding_LatinProp_Exp,100-Atten_Feeding_LatinProp_Exp)~Batch+Treatment,random=~1|Group, family=quasibinomial, data=data)) #ns

wald.test(vcov(mo17),fixef(mo17), Terms=4) #

res_ABT_Feed_Lat<-as.vector(residuals(mo17))

data_ABT_res<-cbind(data_ABT_res,res_ABT_Feed_Lat)

summary(mo18<-glmmPQL(cbind(Loc_Prop_Exp,100-Loc_Prop_Exp)~Batch+Treatment,random=~1|Group, family=quasibinomial, data=data)) #ns

res_ABT_Loc<-as.vector(residuals(mo18))

data_ABT_res<-cbind(data_ABT_res,res_ABT_Loc)

summary(mo19<-glmmPQL(cbind(Expl_Arena_Prop_Exp,100-Expl_Arena_Prop_Exp)~Batch+Treatment,random=~1|Group, family=quasibinomial, data=data)) #ns

wald.test(vcov(mo19),fixef(mo19), Terms=4) #

res_ABT_Expl<-as.vector(residuals(mo19))

res_ABT_Expl_NA<-c(NA,NA,res_ABT_Expl)

data_ABT_res<-cbind(data_ABT_res,res_ABT_Expl_NA)

summary(mo20<-glmmPQL(cbind(Vigi_Prop_Exp,100-Vigi_Prop_Exp)~Batch+Treatment,random=~1|Group, family=quasibinomial, data=data)) #ns

# subset na

data.na<-data[-c(1,2),]

summary(mo20<-glmmPQL(cbind(Vigi_Prop_Exp,100-Vigi_Prop_Exp)~Batch+Treatment,random=~1|Group, family=quasibinomial, data=data.na)) #ns

wald.test(vcov(mo20),fixef(mo20), Terms=4) #

res_ABT_Vigi<-as.vector(residuals(mo20))

res_ABT_Vigi_NA<-c(NA,NA,res_ABT_Vigi)

data_ABT_res<-cbind(data_ABT_res,res_ABT_Vigi_NA)

write.csv(data_ABT_res, file = "ABT_Exp_Res2.csv")

# Table outputs

se <- function(x) sqrt(var(x,na.rm=TRUE)/(length(x)-sum(is.na(x)) ))

cbind(round(apply(data[,c(20:29)],2,mean,na.rm=TRUE),0),round(apply(data[,c(20:29)],2,se),1)) # all table

data_pos<-subset(data,Treatment=="Positive")

data_neg<-subset(data,Treatment=="Negative")

cbind(round(apply(data_pos[,c(20:29)],2,mean,na.rm=TRUE),0),round(apply(data_pos[,c(20:29)],2,se),1)) # pos table

cbind(round(apply(data_neg[,c(20:29)],2,mean,na.rm=TRUE),0),round(apply(data_neg[,c(20:29)],2,se),1)) # neg table

pos.neg<-cbind(round(apply(data_pos[,c(20:29)],2,mean,na.rm=TRUE),0),round(apply(data_pos[,c(20:29)],2,se),1),round(apply(data_neg[,c(20:29)],2,mean,na.rm=TRUE),0),round(apply(data_neg[,c(20:29)],2,se),1))

pos.neg

data_nonfear<-subset(data,Curiousity=="Nonfear")

data_fear<-subset(data,Curiousity=="Fear")

cbind(round(apply(data_fear[,c(20:29)],2,mean,na.rm=TRUE),0),round(apply(data_fear[,c(20:29)],2,se),1))

cbind(round(apply(data_nonfear[,c(20:29)],2,mean,na.rm=TRUE),0),round(apply(data_nonfear[,c(20:29)],2,se),1))

data_soc<-subset(data,Sociability=="Soc")

data_nonsoc<-subset(data,Sociability=="Nonsoc")

cbind(round(apply(data_soc[,c(20:29)],2,mean,na.rm=TRUE),0),round(apply(data_soc[,c(20:29)],2,se),1))

cbind(round(apply(data_nonsoc[,c(20:29)],2,mean,na.rm=TRUE),0),round(apply(data_nonsoc[,c(20:29)],2,se),1))

data_posinact<-subset(data_pos,Activity=="Inact")

data_neginact<-subset(data_neg,Activity=="Inact")

data_negact<-subset(data_neg,Activity=="Act")

cbind(round(apply(data_posinact[,c(20:29)],2,mean,na.rm=TRUE),0),round(apply(data_posinact[,c(20:29)],2,se),1))

cbind(round(apply(data_neginact[,c(20:29)],2,mean,na.rm=TRUE),0),round(apply(data_neginact[,c(20:29)],2,se),1))

cbind(round(apply(data_negact[,c(20:29)],2,mean,na.rm=TRUE),0),round(apply(data_negact[,c(20:29)],2,se),1))

data_posnonsoc<-subset(data_pos,Sociability=="Nonsoc")

data_negnonsoc<-subset(data_neg,Sociability=="Nonsoc")

data_negsoc<-subset(data_neg,Sociability=="Soc")

cbind(round(apply(data_posnonsoc[,c(20:29)],2,mean,na.rm=TRUE),0),round(apply(data_posnonsoc[,c(20:29)],2,se),1))

cbind(round(apply(data_negnonsoc[,c(20:29)],2,mean,na.rm=TRUE),0),round(apply(data_negnonsoc[,c(20:29)],2,se),1))

cbind(round(apply(data_negsoc[,c(20:29)],2,mean,na.rm=TRUE),0),round(apply(data_negsoc[,c(20:29)],2,se),1))

```

**Strategy 1b: investigate the effect of all three housing conditions (only) on ABT responses**

```{r echo=T, results='hide'}

data2<-read.table("/Users/louise/Documents/PhD/Papers/JBT-PT/Rebuttal 1/ABT_3Housing_Manuscript_R1.txt",h=T)

str(data2)

data2$Batch<-as.factor(data2$Batch)

data2$Housing<-as.factor(data2$Housing)

data2$Cow<-as.factor(data2$Cow)

data2$Activity<-as.factor(data2$Activity)

data2$Curiousity<-as.factor(data2$Fearfulness)

data2$Sociability<-as.factor(data2$Sociability)

data2$Group<-as.factor(data2$Group)

nrow(data2) # should be 76

str(data2)

# dataset mean ± se

se <- function(x) sqrt(var(x,na.rm=TRUE)/(length(x)-sum(is.na(x)) ))

data2_conv<-subset(data2,Housing=="Reference")

data2_pos<-subset(data2,Housing=="Positive")

data2_neg<-subset(data2,Housing=="Negative")

cbind(round(apply(data2_conv[,c(5:14)],2,mean,na.rm=TRUE),0),round(apply(data2_conv[,c(5:14)],2,se),1))# conv table

cbind(round(apply(data2_pos[,c(5:14)],2,mean,na.rm=TRUE),0),round(apply(data2_pos[,c(5:14)],2,se),1)) # pos table

cbind(round(apply(data2_neg[,c(5:14)],2,mean,na.rm=TRUE),0),round(apply(data2_neg[,c(5:14)],2,se),1)) # neg table

# Rebuttal 1 - ABD score

summary(moABD<-lme4::lmer(ABD_Prop~Batch+Housing+(1|Group/Cow),data=data2))

wald.test(vcov(moABD),fixef(moABD), Terms=4:5) #s

model_obj_em<-emmeans(moABD, ~ Housing)

summary(pairs(model_obj_em),adjust="none") # ABD_prop decreases in both experimental conditions compared with REF

# Time spent looking at the threat

summary(mo11<-glmmPQL(cbind(Atten_Threat_Prop,100-Atten_Threat_Prop)~Batch+Housing,random=~1|Group/Cow, family=quasibinomial, data=data2))

wald.test(vcov(mo11),fixef(mo11), Terms=4:5) #s

model_obj_em<-emmeans(mo11, ~ Housing)

summary(pairs(model_obj_em),adjust="none") # conventional positive *3

# Time spent eating

summary(mo13<-glmmPQL(cbind(Feeding_Prop,100-Feeding_Prop)~Batch+Housing,random=~1|Group/Cow, family=quasibinomial, data=data2))

wald.test(vcov(mo13),fixef(mo13), Terms=4:5) #s

model_obj_em<-emmeans(mo13, ~ Housing)

summary(pairs(model_obj_em),adjust="none") # conv-neg and conv-pos

# Relative positive attention

summary(mo14<-glmmPQL(cbind(Positive_Diff_Att,100-Positive_Diff_Att)~Batch+Housing,random=~1|Group/Cow, family=quasibinomial, data=data2))

wald.test(vcov(mo14),fixef(mo14), Terms=4:5) #s

model_obj_em<-emmeans(mo14, ~ Housing)

summary(pairs(model_obj_em),adjust="none") # conv

# Latency to look at the threat

summary(mo15<-glmmPQL(cbind(Atten_Threat_LatinProp,100-Atten_Threat_LatinProp)~Batch+Housing,random=~1|Group/Cow, family=quasibinomial, data=data2))

wald.test(vcov(mo15),fixef(mo15), Terms=4:5) #s

model_obj_em<-emmeans(mo15, ~ Housing)

summary(pairs(model_obj_em),adjust="none")

## Rebuttal 1 - Latency to look at the threat (pull apart Activity effect from individual variation)

summary(mo15r<-glmmPQL(cbind(Atten_Threat_LatinProp,100-Atten_Threat_LatinProp)~Batch+Housing+Activity+Housing:Activity,random=~1|Group/Cow, family=quasibinomial, data=data2))

wald.test(vcov(mo15r),fixef(mo15r), Terms=4:5) #

wald.test(vcov(mo15r),fixef(mo15r), Terms=6) #

wald.test(vcov(mo15r),fixef(mo15r), Terms=7:8) # 0.72 ns

model_obj_em<-emmeans(mo15r, ~ Housing:Activity)

my_pvalues<-cbind(as.data.frame(emmeans(mo15r, list(pairwise ~ Housing:Activity), adjust = "none")$`pairwise differences of Housing, Activity`)[,c(1:6)])

my_adjp.CI.HR<-cbind(my_pvalues,adj_p=my_pvalues$p.value*9,CI_Inf=my_pvalues$estimate-1.96*my_pvalues$SE,CI_Sup=my_pvalues$estimate+1.96*my_pvalues$SE) #

my_select.adjp.CI.HR<-my_adjp.CI.HR[c(1,2,3,6,8,12,13,14,15),]

my_select.adjp.CI.HR # ns

summary(mo15r2<-glmmPQL(cbind(Atten_Threat_LatinProp,100-Atten_Threat_LatinProp)~Batch+Housing+Activity,random=~1|Group/Cow, family=quasibinomial, data=data2))

wald.test(vcov(mo15r),fixef(mo15r), Terms=4:5) #

wald.test(vcov(mo15r),fixef(mo15r), Terms=6) # no effect of activity

model_obj_em<-emmeans(mo15r, ~ Housing:Activity)

my_pvalues<-cbind(as.data.frame(emmeans(mo15r, list(pairwise ~ Housing:Activity), adjust = "none")$`pairwise differences of Housing, Activity`)[,c(1:6)])

my_adjp.CI.HR<-cbind(my_pvalues,adj_p=my_pvalues$p.value*9,CI_Inf=my_pvalues$estimate-1.96*my_pvalues$SE,CI_Sup=my_pvalues$estimate+1.96*my_pvalues$SE) #

my_select.adjp.CI.HR<-my_adjp.CI.HR[c(1,2,3,6,8,12,13,14,15),]

my_select.adjp.CI.HR # ns

## Rebuttal 1 - Latency to look at the threat (pull apart Sociability effect from individual variation)

summary(mo15r<-glmmPQL(cbind(Atten_Threat_LatinProp,100-Atten_Threat_LatinProp)~Batch+Housing+Sociability+Housing:Sociability,random=~1|Group/Cow, family=quasibinomial, data=data2))

wald.test(vcov(mo15r),fixef(mo15r), Terms=4:5) #

wald.test(vcov(mo15r),fixef(mo15r), Terms=6) #

wald.test(vcov(mo15r),fixef(mo15r), Terms=7:8) # 0.29 ns

model_obj_em<-emmeans(mo15r, ~ Housing:Sociability)

my_pvalues<-cbind(as.data.frame(emmeans(mo15r, list(pairwise ~ Housing:Sociability), adjust = "none")$`pairwise differences of Housing, Sociability`)[,c(1:6)])

my_adjp.CI.HR<-cbind(my_pvalues,adj_p=my_pvalues$p.value*9,CI_Inf=my_pvalues$estimate-1.96*my_pvalues$SE,CI_Sup=my_pvalues$estimate+1.96*my_pvalues$SE) #

my_select.adjp.CI.HR<-my_adjp.CI.HR[c(1,2,3,6,8,12,13,14,15),]

my_select.adjp.CI.HR # ns

# Latency to eat

summary(mo17<-glmmPQL(cbind(Atten_Feeding_LatinProp,100-Atten_Feeding_LatinProp)~Batch+Housing,random=~1|Group/Cow, family=quasibinomial, data=data2)) #

wald.test(vcov(mo17),fixef(mo17), Terms=4:5) #s

model_obj_em<-emmeans(mo17, ~ Housing)

summary(pairs(model_obj_em),adjust="none")

# Locomotion

summary(mo18<-glmmPQL(cbind(Loc_Prop,100-Loc_Prop)~Batch+Housing,random=~1|Group/Cow, family=quasibinomial, data=data2)) # neg

wald.test(vcov(mo18),fixef(mo18), Terms=4:5) #s

model_obj_em<-emmeans(mo18, ~ Housing)

summary(pairs(model_obj_em),adjust="none")

# Time in proximity with walls

summary(mo19<-glmmPQL(cbind(Expl_Arena_Prop,100-Expl_Arena_Prop)~Batch+Housing,random=~1|Group/Cow, family=quasibinomial, data=data2)) #

wald.test(vcov(mo19),fixef(mo19), Terms=4:5) #ns

model_obj_em<-emmeans(mo19, ~ Housing)

summary(pairs(model_obj_em),adjust="none")

# Time head up

summary(mo20<-glmmPQL(cbind(Vigi_Prop,100-Vigi_Prop)~Batch+Housing,random=~1|Group/Cow, family=quasibinomial, data=data2))

wald.test(vcov(mo20),fixef(mo20), Terms=4:5) # tend

model_obj_em<-emmeans(mo20, ~ Housing)

summary(pairs(model_obj_em),adjust="none")

```

**Strategy 2: account for individual differences (of all types including potentially PT effect) using responses under the reference conditions as covariates**

```{r}

data<-read.table("/Users/louise/Documents/PhD/Papers/JBT-PT/Rebuttal 1/ABT_Exp_Manuscript_R1.txt",h=T)

data$Batch<-as.factor(data$Batch)

data$Treatment<-as.factor(data$Treatment)

data$Cow<-as.factor(data$Cow)

data$Activity<-as.factor(data$Activity)

data$Curiousity<-as.factor(data$Fearfulness)

data$Sociability<-as.factor(data$Sociability)

data$Group<-as.factor(data$Group)

nrow(data) # should be 38

str(data)

# logit function

logit <- function(x) log((x+0.5)/(100-x+0.5))

# Rebuttal 1 - ABD score

moABD<-lmer(ABD_Prop_Exp~Batch+Treatment+ABD_Prop_Ref+ABD_Prop_Ref:Treatment+(1|Group),data=data) #ns

wald.test(vcov(moABD),fixef(moABD), Terms=4) # 0.74

wald.test(vcov(moABD),fixef(moABD), Terms=5) # 0.046

wald.test(vcov(moABD),fixef(moABD), Terms=6) # 0.28 no interaction

plot(data$ABD_Prop_Exp,data$ABD_Prop_Ref)

moABD<-lmer(ABD_Prop_Exp~Batch+Treatment+ABD_Prop_Ref+(1|Group),data=data) #ns

wald.test(vcov(moABD),fixef(moABD), Terms=4) # 0.74

wald.test(vcov(moABD),fixef(moABD), Terms=5) # 0.093

# Put the covariate on the logit scale

data$Logit_Atten_Threat_Prop_Conv<-logit(data$Atten_Threat_Prop_Ref)

data$Logit_Atten_Bucket_Prop_Conv<-logit(data$Atten_Bucket_Prop_Ref)

data$Logit_Feeding_Prop_Conv<-logit(data$Feeding_Prop_Ref)

data$Logit_Positive_Diff_Att_Conv<-logit(data$Positive_Diff_Att_Ref)

data$Logit_Loc_Prop_Conv<-logit(data$Loc_Prop_Ref)

data$Logit_Expl_Arena_Prop_Conv<-logit(data$Expl_Arena_Prop_Ref)

data$Logit_Vigi_Prop_Conv<-logit(data$Vigi_Prop_Ref)

data$Logit_Atten_Threat_LatinProp_Conv<-logit(data$Atten_Threat_LatinProp_Ref)

data$Logit_Atten_Bucket_LatinProp_Conv<-logit(data$Atten_Bucket_LatinProp_Ref)

data$Logit_Atten_Feeding_LatinProp_Conv<-logit(data$Atten_Feeding_LatinProp_Ref)

# Proportion to look at the threat

summary(mo11<-glmmPQL(cbind(Atten_Threat_Prop_Exp,100-Atten_Threat_Prop_Exp)~Batch+Treatment+Logit_Atten_Threat_Prop_Conv+Logit_Atten_Threat_Prop_Conv:Treatment,random=~1|Group, family=quasibinomial, data=data)) #ns

nrow(data)

wald.test(vcov(mo11),fixef(mo11), Terms=4) #

wald.test(vcov(mo11),fixef(mo11), Terms=5) #

wald.test(vcov(mo11),fixef(mo11), Terms=6) # ns (0.34)

summary(mo1f<-glmmPQL(cbind(Atten_Threat_Prop_Exp,100-Atten_Threat_Prop_Exp)~Batch+Treatment+Logit_Atten_Threat_Prop_Conv,random=~1|Group, family=quasibinomial, data=data)) #ns

nrow(data)

wald.test(vcov(mo1f),fixef(mo1f), Terms=4) #

wald.test(vcov(mo1f),fixef(mo1f), Terms=5) #

# Proportion to eat

summary(mo21<-glmmPQL(cbind(Feeding_Prop_Exp,100-Feeding_Prop_Exp)~Batch+Treatment+Logit_Feeding_Prop_Conv+Treatment:Logit_Feeding_Prop_Conv,random=~1|Group, family=quasibinomial, data=data)) # tendency effect logit_feed

wald.test(vcov(mo21),fixef(mo21), Terms=4) #

wald.test(vcov(mo21),fixef(mo21), Terms=5) #

wald.test(vcov(mo21),fixef(mo21), Terms=6) # ns

summary(mo2f<-glmmPQL(cbind(Feeding_Prop_Exp,100-Feeding_Prop_Exp)~Batch+Treatment+Logit_Feeding_Prop_Conv,random=~1|Group, family=quasibinomial, data=data)) # effect logit_feed

wald.test(vcov(mo2f),fixef(mo2f), Terms=4) #

wald.test(vcov(mo2f),fixef(mo2f), Terms=5) #

# Relative positive attention

summary(mo31<-glmmPQL(cbind(Positive_Diff_Att_Exp,100-Positive_Diff_Att_Exp)~Batch+Treatment+Logit_Positive_Diff_Att_Conv+Logit_Positive_Diff_Att_Conv:Treatment,random=~1|Group, family=quasibinomial, data=data)) #

wald.test(vcov(mo31),fixef(mo31), Terms=4) #

wald.test(vcov(mo31),fixef(mo31), Terms=5) #

wald.test(vcov(mo31),fixef(mo31), Terms=6) #

summary(mo31<-glmmPQL(cbind(Positive_Diff_Att_Exp,100-Positive_Diff_Att_Exp)~Batch+Treatment++Logit_Positive_Diff_Att_Conv:Treatment,random=~1|Group, family=quasibinomial, data=data)) #

summary(mo3f<-glmmPQL(cbind(Positive_Diff_Att_Exp,100-Positive_Diff_Att_Exp)~Batch+Treatment+Logit_Positive_Diff_Att_Conv,random=~1|Group, family=quasibinomial, data=data))

wald.test(vcov(mo3f),fixef(mo3f), Terms=4) #

wald.test(vcov(mo3f),fixef(mo3f), Terms=5) #

# Lat to look at the threat

summary(mo41<-glmmPQL(cbind(Atten_Threat_LatinProp_Exp,100-Atten_Threat_LatinProp_Exp)~Batch+Treatment+Logit_Atten_Threat_LatinProp_Conv+Logit_Atten_Threat_LatinProp_Conv:Treatment,random=~1|Group, family=quasibinomial, data=data)) # ns

wald.test(vcov(mo41),fixef(mo41), Terms=4) #

wald.test(vcov(mo41),fixef(mo41), Terms=5) #

wald.test(vcov(mo41),fixef(mo41), Terms=6) #

summary(mo4f<-glmmPQL(cbind(Atten_Threat_LatinProp_Exp,100-Atten_Threat_LatinProp_Exp)~Batch+Treatment+Logit_Atten_Threat_LatinProp_Conv,random=~1|Group, family=quasibinomial, data=data)) # ns but treatment effect

wald.test(vcov(mo4f),fixef(mo4f), Terms=4) #

wald.test(vcov(mo4f),fixef(mo4f), Terms=5) #

# Lat to eat

summary(mo51<-glmmPQL(cbind(Atten_Feeding_LatinProp_Exp,100-Atten_Feeding_LatinProp_Exp)~Batch+Treatment+Logit_Atten_Feeding_LatinProp_Conv+Logit_Atten_Feeding_LatinProp_Conv:Treatment,random=~1|Group, family=quasibinomial, data=data)) # effect logit!

wald.test(vcov(mo51),fixef(mo51), Terms=4) #

wald.test(vcov(mo51),fixef(mo51), Terms=5) #

wald.test(vcov(mo51),fixef(mo51), Terms=6) #

summary(mo51<-glmmPQL(cbind(Atten_Feeding_LatinProp_Exp,100-Atten_Feeding_LatinProp_Exp)~Batch+Treatment+Logit_Atten_Feeding_LatinProp_Conv,random=~1|Group, family=quasibinomial, data=data)) # effect logit!

wald.test(vcov(mo51),fixef(mo51), Terms=4) #

wald.test(vcov(mo51),fixef(mo51), Terms=5) #

# Time spent in locomotion

summary(mo61<-glmmPQL(cbind(Loc_Prop_Exp,100-Loc_Prop_Exp)~Batch+Treatment+Logit_Loc_Prop_Conv+Logit_Loc_Prop_Conv:Treatment,random=~1|Group, family=quasibinomial, data=data)) #ns tendency effect logit

wald.test(vcov(mo61),fixef(mo61), Terms=4) #

wald.test(vcov(mo61),fixef(mo61), Terms=5) #

wald.test(vcov(mo61),fixef(mo61), Terms=6) #

summary(mo61<-glmmPQL(cbind(Loc_Prop_Exp,100-Loc_Prop_Exp)~Batch+Treatment+Logit_Loc_Prop_Conv,random=~1|Group, family=quasibinomial, data=data)) #ns tendency effect logit

wald.test(vcov(mo61),fixef(mo61), Terms=4) #

wald.test(vcov(mo61),fixef(mo61), Terms=5) #

# Time spent in contact with the walls

summary(mo29<-glmmPQL(cbind(Expl_Arena_Prop_Exp,100-Expl_Arena_Prop_Exp)~Batch+Treatment+Logit_Expl_Arena_Prop_Conv+Logit_Expl_Arena_Prop_Conv:Treatment,random=~1|Group, family=quasibinomial, data=data)) #ns

wald.test(vcov(mo29),fixef(mo29), Terms=4) #

wald.test(vcov(mo29),fixef(mo29), Terms=5) #

wald.test(vcov(mo29),fixef(mo29), Terms=6) #

summary(mo29b<-glmmPQL(cbind(Expl_Arena_Prop_Exp,100-Expl_Arena_Prop_Exp)~Batch+Treatment+Logit_Expl_Arena_Prop_Conv:Treatment,random=~1|Group, family=quasibinomial, data=data)) #ns

summary(mo29<-glmmPQL(cbind(Expl_Arena_Prop_Exp,100-Expl_Arena_Prop_Exp)~Batch+Treatment+Logit_Expl_Arena_Prop_Conv,random=~1|Group, family=quasibinomial, data=data)) #ns

wald.test(vcov(mo29),fixef(mo29), Terms=4) #

wald.test(vcov(mo29),fixef(mo29), Terms=5) #

# Time spend in contact with head up

summary(mo30<-glmmPQL(cbind(Vigi_Prop_Exp,100-Vigi_Prop_Exp)~Batch+Treatment+Logit_Vigi_Prop_Conv+Logit_Vigi_Prop_Conv:Treatment,random=~1|Group, family=quasibinomial, data=data)) # interaction

wald.test(vcov(mo30),fixef(mo30), Terms=4) #

wald.test(vcov(mo30),fixef(mo30), Terms=5) #

wald.test(vcov(mo30),fixef(mo30), Terms=6) #

summary(mo30<-glmmPQL(cbind(Vigi_Prop_Exp,100-Vigi_Prop_Exp)~Batch+Treatment+Logit_Vigi_Prop_Conv:Treatment,random=~1|Group, family=quasibinomial, data=data)) # interaction

wald.test(vcov(mo30),fixef(mo30), Terms=5) #neg

wald.test(vcov(mo30),fixef(mo30), Terms=6) #pos

data_pos<-subset(data,Treatment=="Positive")

summary(mo30<-glmmPQL(cbind(Vigi_Prop_Exp,100-Vigi_Prop_Exp)~Batch+Logit_Vigi_Prop_Conv,random=~1|Group, family=quasibinomial, data=data_pos)) #

wald.test(vcov(mo30),fixef(mo30), Terms=4) #

data_neg<-subset(data,Treatment=="Negative")

summary(mo30<-glmmPQL(cbind(Vigi_Prop_Exp,100-Vigi_Prop_Exp)~Batch+Logit_Vigi_Prop_Conv,random=~1|Group, family=quasibinomial, data=data_neg)) #

wald.test(vcov(mo30),fixef(mo30), Terms=4) #

```

**Strategy 2b: With the levels of treatment**

```{r echo=T, results='hide'}

# Rebuttal 1 - ABD Score summary(moABD<-lmer(ABD_Prop_Exp~Batch+Treatment:ABD_Prop_Ref+(1|Group),data=data)) #ns

summary(mo21<-glmmPQL(cbind(Atten_Threat_Prop_Exp,100-Atten_Threat_Prop_Exp)~Batch+Treatment+Logit_Atten_Threat_Prop_Conv:Treatment,random=~1|Group, family=quasibinomial, data=data)) #ns

summary(mo23<-glmmPQL(cbind(Feeding_Prop_Exp,100-Feeding_Prop_Exp)~Batch+Treatment++Treatment:Logit_Feeding_Prop_Conv,random=~1|Group, family=quasibinomial, data=data)) # tendency effect logit_feed for negative treatment

summary(mo24<-glmmPQL(cbind(Positive_Diff_Att_Exp,100-Positive_Diff_Att_Exp)~Batch+Treatment+Logit_Positive_Diff_Att_Conv:Treatment,random=~1|Group, family=quasibinomial, data=data)) # tendency effect for neg treatment

summary(mo25<-glmmPQL(cbind(Atten_Threat_LatinProp_Exp,100-Atten_Threat_LatinProp_Exp)~Batch+Treatment+Logit_Atten_Threat_LatinProp_Conv:Treatment,random=~1|Group, family=quasibinomial, data=data)) # ns

summary(mo27<-glmmPQL(cbind(Atten_Feeding_LatinProp_Exp,100-Atten_Feeding_LatinProp_Exp)~Batch+Treatment+Logit_Atten_Feeding_LatinProp_Conv:Treatment,random=~1|Group, family=quasibinomial, data=data)) # effect logit! pos and neg

summary(mo28<-glmmPQL(cbind(Loc_Prop_Exp,100-Loc_Prop_Exp)~Batch+Treatment+Logit_Loc_Prop_Conv:Treatment,random=~1|Group, family=quasibinomial, data=data)) #ns tendency effect logit neg and pos

summary(mo29<-glmmPQL(cbind(Expl_Arena_Prop_Exp,100-Expl_Arena_Prop_Exp)~Batch+Treatment+Logit_Expl_Arena_Prop_Conv:Treatment,random=~1|Group, family=quasibinomial, data=data)) #ns

summary(mo30<-glmmPQL(cbind(Vigi_Prop_Exp,100-Vigi_Prop_Exp)~Batch+Treatment+Logit_Vigi_Prop_Conv:Treatment,random=~1|Group, family=quasibinomial, data=data)) # Pos effect

wald.test(vcov(mo30),fixef(mo30), Terms=5) #

wald.test(vcov(mo30),fixef(mo30), Terms=6) #

```

**Strategy 3: exploratory analyses to investigate the influence of personality traits on the ABT responses**

From there, the idea is to write a code that runs all the potential models and select the best one.

#### **Transverse approach**

```{r echo=T, results='hide'}

# pb with Treatment:Activity:Sociability and Activity:Curiousity:Sociability

# Rebuttal 1 - ABD score

## S1

summary(moABD<-lmer(ABD_Prop_Exp~Batch+Treatment+Activity+Curiousity+Sociability+Treatment:Activity+Treatment:Curiousity+Treatment:Sociability+Activity:Curiousity+Activity:Sociability+Curiousity:Sociability+Treatment:Activity:Curiousity+Treatment:Curiousity:Sociability+(1|Group), data=data))

wald.test(vcov(moABD),fixef(moABD), Terms=14) #

wald.test(vcov(moABD),fixef(moABD), Terms=15) #

## S2

summary(moABD<-lmer(ABD_Prop_Exp~Batch+Treatment+Activity+Curiousity+Sociability+Treatment:Activity+Treatment:Curiousity+Treatment:Sociability+Activity:Curiousity+Activity:Sociability+Curiousity:Sociability+(1|Group), data=data))

wald.test(vcov(moABD),fixef(moABD), Terms=11) #

wald.test(vcov(moABD),fixef(moABD), Terms=12) #

wald.test(vcov(moABD),fixef(moABD), Terms=13) #

## S3

summary(moABD<-lmer(ABD_Prop_Exp~Batch+Treatment+Activity+Curiousity+Sociability+Treatment:Activity+Treatment:Curiousity+Treatment:Sociability+(1|Group), data=data))

wald.test(vcov(moABD),fixef(moABD), Terms=8) #

wald.test(vcov(moABD),fixef(moABD), Terms=9) #

wald.test(vcov(moABD),fixef(moABD), Terms=10) #

## S4 - final

summary(moABD<-lmer(ABD_Prop_Exp~Batch+Treatment+Activity+Curiousity+Sociability+(1|Group), data=data))

wald.test(vcov(moABD),fixef(moABD), Terms=4) #

wald.test(vcov(moABD),fixef(moABD), Terms=5) #

wald.test(vcov(moABD),fixef(moABD), Terms=6) # tendency for sociability

# Prop attention to threat

## S1

summary(mo31<-glmmPQL(cbind(Atten_Threat_Prop_Exp,100-Atten_Threat_Prop_Exp)~Batch+Treatment+Activity+Curiousity+Sociability+Treatment:Activity+Treatment:Curiousity+Treatment:Sociability+Activity:Curiousity+Activity:Sociability+Curiousity:Sociability+Treatment:Activity:Curiousity+Treatment:Curiousity:Sociability,random=~1|Group, family=quasibinomial, data=data))

wald.test(vcov(mo31),fixef(mo31), Terms=14) #

wald.test(vcov(mo31),fixef(mo31), Terms=15) #

## S1 - B

summary(mo31<-glmmPQL(cbind(Atten_Threat_Prop_Exp,100-Atten_Threat_Prop_Exp)~Batch+Treatment+Activity+Curiousity+Sociability+Treatment:Activity+Treatment:Curiousity+Treatment:Sociability+Activity:Curiousity+Activity:Sociability+Curiousity:Sociability,random=~1|Group, family=quasibinomial, data=data))

wald.test(vcov(mo31),fixef(mo31), Terms=11) #

wald.test(vcov(mo31),fixef(mo31), Terms=12) #

wald.test(vcov(mo31),fixef(mo31), Terms=13) #

## S1 - C

summary(mo31<-glmmPQL(cbind(Atten_Threat_Prop_Exp,100-Atten_Threat_Prop_Exp)~Batch+Treatment+Activity+Curiousity+Sociability+Treatment:Activity+Treatment:Curiousity+Treatment:Sociability+Curiousity:Sociability,random=~1|Group, family=quasibinomial, data=data))

wald.test(vcov(mo31),fixef(mo31), Terms=8) #

wald.test(vcov(mo31),fixef(mo31), Terms=9) #

wald.test(vcov(mo31),fixef(mo31), Terms=10) #

wald.test(vcov(mo31),fixef(mo31), Terms=11) #

## S3 MODEL FINAL

summary(mo31_f<-glmmPQL(cbind(Atten_Threat_Prop_Exp,100-Atten_Threat_Prop_Exp)~Batch+Treatment+Activity+Curiousity+Sociability+Treatment:Activity+Curiousity:Sociability,random=~1|Group, family=quasibinomial, data=data))

wald.test(vcov(mo31_f),fixef(mo31_f), Terms=1:3)

wald.test(vcov(mo31_f),fixef(mo31_f), Terms=4)

wald.test(vcov(mo31_f),fixef(mo31_f), Terms=5)

wald.test(vcov(mo31_f),fixef(mo31_f), Terms=6)

wald.test(vcov(mo31_f),fixef(mo31_f), Terms=7)

wald.test(vcov(mo31_f),fixef(mo31_f), Terms=8) #s

wald.test(vcov(mo31_f),fixef(mo31_f), Terms=9) #s

model_obj_em<-emmeans(mo31_f, ~ Treatment:Activity)

summary(pairs(model_obj_em),adjust="none") # *4

# neg,Act - pos,Act 1.0981 0.582 8 1.887 0.0959

# neg,Act - neg,Inact 2.0521 0.927 21 2.214 0.0380

# pos,Act - pos,Inact -0.0833 0.599 21 -0.139 0.8908

# neg,Inact - pos,Inact -1.0373 0.938 8 -1.106 0.3007

model_obj_em1<-emmeans(mo31_f, ~ Curiousity:Sociability)

summary(pairs(model_obj_em1),adjust="none") # *4

# contrast estimate SE df t.ratio p.value

# Cur,Nonsoc - Noncur,Nonsoc -1.483 0.777 21 -1.908 0.0702

# Cur,Nonsoc - Cur,Soc -1.720 0.776 21 -2.217 0.0378

# Cur,Nonsoc - Noncur,Soc -0.912 0.825 21 -1.106 0.2812

# Noncur,Nonsoc - Cur,Soc -0.237 0.459 21 -0.516 0.6114

# Noncur,Nonsoc - Noncur,Soc 0.571 0.605 21 0.943 0.3563

# Cur,Soc - Noncur,Soc 0.808 0.613 21 1.317 0.2019

summary(mo31_f<-glmmPQL(cbind(Atten_Threat_Prop_Exp,100-Atten_Threat_Prop_Exp)~Batch+Treatment+Activity+Curiousity+Sociability,random=~1|Group, family=quasibinomial, data=data))

# Prop feeding

## S1

summary(mo33<-glmmPQL(cbind(Feeding_Prop_Exp,100-Feeding_Prop_Exp)~Batch+Treatment+Activity+Curiousity+Sociability+Treatment:Activity+Treatment:Curiousity+Treatment:Sociability+Activity:Curiousity+Activity:Sociability+Curiousity:Sociability+Treatment:Activity:Curiousity+Treatment:Curiousity:Sociability,random=~1|Group, family=quasibinomial, data=data))

wald.test(vcov(mo33),fixef(mo33), Terms=14) #

wald.test(vcov(mo33),fixef(mo33), Terms=15) #

## S2

summary(mo33_2<-glmmPQL(cbind(Feeding_Prop_Exp,100-Feeding_Prop_Exp)~Batch+Treatment+Activity+Curiousity+Sociability+Treatment:Activity+Treatment:Curiousity+Treatment:Sociability+Activity:Curiousity+Activity:Sociability+Curiousity:Sociability,random=~1|Group, family=quasibinomial, data=data))

wald.test(vcov(mo33_2),fixef(mo33_2), Terms=11)

wald.test(vcov(mo33_2),fixef(mo33_2), Terms=12) #

wald.test(vcov(mo33_2),fixef(mo33_2), Terms=13) #

## S3

summary(mo33_3<-glmmPQL(cbind(Feeding_Prop_Exp,100-Feeding_Prop_Exp)~Batch+Treatment+Activity+Curiousity+Sociability+Treatment:Activity+Treatment:Curiousity+Treatment:Sociability,random=~1|Group, family=quasibinomial, data=data))

wald.test(vcov(mo33_3),fixef(mo33_3), Terms=8)

wald.test(vcov(mo33_3),fixef(mo33_3), Terms=9) #

wald.test(vcov(mo33_3),fixef(mo33_3), Terms=10) #

## S4 MODEL FINAL

summary(mo33_f<-glmmPQL(cbind(Feeding_Prop_Exp,100-Feeding_Prop_Exp)~Batch+Treatment+Activity+Curiousity+Sociability,random=~1|Group, family=quasibinomial, data=data))

wald.test(vcov(mo33_f),fixef(mo33_f), Terms=4)

wald.test(vcov(mo33_f),fixef(mo33_f), Terms=5)

wald.test(vcov(mo33_f),fixef(mo33_f), Terms=6)

wald.test(vcov(mo33_f),fixef(mo33_f), Terms=7)

# Relative Positive Diff Att

## S1

summary(mo34<-glmmPQL(cbind(Positive_Diff_Att_Exp,100-Positive_Diff_Att_Exp)~Batch+Treatment+Activity+Curiousity+Sociability+Treatment:Activity+Treatment:Curiousity+Treatment:Sociability+Activity:Curiousity+Activity:Sociability+Curiousity:Sociability+Treatment:Activity:Curiousity+Treatment:Curiousity:Sociability,random=~1|Group, family=quasibinomial, data=data))

wald.test(vcov(mo34),fixef(mo34), Terms=14) #

wald.test(vcov(mo34),fixef(mo34), Terms=15) #

## S2

summary(mo34_1<-glmmPQL(cbind(Positive_Diff_Att_Exp,100-Positive_Diff_Att_Exp)~Batch+Treatment+Activity+Curiousity+Sociability+Treatment:Activity+Treatment:Curiousity+Treatment:Sociability+Activity:Curiousity+Activity:Sociability+Curiousity:Sociability,random=~1|Group, family=quasibinomial, data=data))

wald.test(vcov(mo34_1),fixef(mo34_1), Terms=11) #

wald.test(vcov(mo34_1),fixef(mo34_1), Terms=12) #

wald.test(vcov(mo34_1),fixef(mo34_1), Terms=13) #

## S2-B

summary(mo34_1<-glmmPQL(cbind(Positive_Diff_Att_Exp,100-Positive_Diff_Att_Exp)~Batch+Treatment+Activity+Curiousity+Sociability+Treatment:Activity+Treatment:Curiousity+Treatment:Sociability+Activity:Sociability+Curiousity:Sociability,random=~1|Group, family=quasibinomial, data=data))

wald.test(vcov(mo34_1),fixef(mo34_1), Terms=8)

wald.test(vcov(mo34_1),fixef(mo34_1), Terms=9)

wald.test(vcov(mo34_1),fixef(mo34_1), Terms=10)

wald.test(vcov(mo34_1),fixef(mo34_1), Terms=11)

wald.test(vcov(mo34_1),fixef(mo34_1), Terms=12)

## S3-B

summary(mo34_1<-glmmPQL(cbind(Positive_Diff_Att_Exp,100-Positive_Diff_Att_Exp)~Batch+Treatment+Activity+Curiousity+Sociability+Treatment:Activity+Activity:Sociability,random=~1|Group, family=quasibinomial, data=data))

wald.test(vcov(mo34_1),fixef(mo34_1), Terms=8)

wald.test(vcov(mo34_1),fixef(mo34_1), Terms=9)

## S3-C1

summary(mo34_1<-glmmPQL(cbind(Positive_Diff_Att_Exp,100-Positive_Diff_Att_Exp)~Batch+Treatment+Activity+Curiousity+Sociability+Treatment:Activity,random=~1|Group, family=quasibinomial, data=data))

wald.test(vcov(mo34_1),fixef(mo34_1), Terms=8)

## S3-C2

summary(mo34_1<-glmmPQL(cbind(Positive_Diff_Att_Exp,100-Positive_Diff_Att_Exp)~Batch+Treatment+Activity+Curiousity+Sociability+Activity:Sociability,random=~1|Group, family=quasibinomial, data=data))

wald.test(vcov(mo34_1),fixef(mo34_1), Terms=8)

## S3

summary(mo34_1<-glmmPQL(cbind(Positive_Diff_Att_Exp,100-Positive_Diff_Att_Exp)~Batch+Treatment+Activity+Curiousity+Sociability+Treatment:Activity+Treatment:Curiousity+Treatment:Sociability+Activity:Sociability+Curiousity:Sociability,random=~1|Group, family=quasibinomial, data=data))

## S4 - A1

summary(mo34_1<-glmmPQL(cbind(Positive_Diff_Att_Exp,100-Positive_Diff_Att_Exp)~Batch+Treatment+Activity+Curiousity+Sociability+Treatment:Activity+Treatment:Curiousity+Treatment:Sociability+Activity:Sociability,random=~1|Group, family=quasibinomial, data=data))

## S4 - A2

summary(mo34_1<-glmmPQL(cbind(Positive_Diff_Att_Exp,100-Positive_Diff_Att_Exp)~Batch+Treatment+Activity+Curiousity+Sociability+Treatment:Activity+Treatment:Curiousity+Treatment:Sociability,random=~1|Group, family=quasibinomial, data=data))

## S4 - A3

summary(mo34_1<-glmmPQL(cbind(Positive_Diff_Att_Exp,100-Positive_Diff_Att_Exp)~Batch+Treatment+Activity+Curiousity+Sociability,random=~1|Group, family=quasibinomial, data=data))

## S4 - B1

summary(mo34_1<-glmmPQL(cbind(Positive_Diff_Att_Exp,100-Positive_Diff_Att_Exp)~Batch+Treatment+Activity+Curiousity+Sociability+Treatment:Activity+Activity:Sociability+Curiousity:Sociability,random=~1|Group, family=quasibinomial, data=data))

## S4 - B2

summary(mo34_1<-glmmPQL(cbind(Positive_Diff_Att_Exp,100-Positive_Diff_Att_Exp)~Batch+Treatment+Activity+Curiousity+Sociability,random=~1|Group, family=quasibinomial, data=data))

## S4 - C1

summary(mo34_1<-glmmPQL(cbind(Positive_Diff_Att_Exp,100-Positive_Diff_Att_Exp)~Batch+Treatment+Activity+Curiousity+Sociability+Treatment:Activity+Activity:Sociability,random=~1|Group, family=quasibinomial, data=data))

## S4 - C2

summary(mo34_1<-glmmPQL(cbind(Positive_Diff_Att_Exp,100-Positive_Diff_Att_Exp)~Batch+Treatment+Activity+Curiousity+Sociability,random=~1|Group, family=quasibinomial, data=data))

## MODEL FINAL WHICHEVER PATHWAY TAKEN

summary(mo34_f<-glmmPQL(cbind(Positive_Diff_Att_Exp,100-Positive_Diff_Att_Exp)~Batch+Treatment+Activity+Curiousity+Sociability,random=~1|Group, family=quasibinomial, data=data))

wald.test(vcov(mo34_f),fixef(mo34_f), Terms=1:3)

wald.test(vcov(mo34_f),fixef(mo34_f), Terms=4)

wald.test(vcov(mo34_f),fixef(mo34_f), Terms=5)

wald.test(vcov(mo34_f),fixef(mo34_f), Terms=6) #s

wald.test(vcov(mo34_f),fixef(mo34_f), Terms=7) #s

# Latency to look at the threat

## S1

summary(mo35<-glmmPQL(cbind(Atten_Threat_LatinProp_Exp,100-Atten_Threat_LatinProp_Exp)~Batch+Treatment+Activity+Curiousity+Sociability+Treatment:Activity+Treatment:Curiousity+Treatment:Sociability+Activity:Curiousity+Activity:Sociability+Curiousity:Sociability+Treatment:Activity:Curiousity+Treatment:Curiousity:Sociability,random=~1|Group, family=quasibinomial, data=data))

wald.test(vcov(mo35),fixef(mo35), Terms=14) #

wald.test(vcov(mo35),fixef(mo35), Terms=15) #

## S2

summary(mo35<-glmmPQL(cbind(Atten_Threat_LatinProp_Exp,100-Atten_Threat_LatinProp_Exp)~Batch+Treatment+Activity+Curiousity+Sociability+Treatment:Activity+Treatment:Curiousity+Treatment:Sociability+Activity:Curiousity+Activity:Sociability+Curiousity:Sociability,random=~1|Group, family=quasibinomial, data=data))

wald.test(vcov(mo35),fixef(mo35), Terms=11) #

wald.test(vcov(mo35),fixef(mo35), Terms=12) #

wald.test(vcov(mo35),fixef(mo35), Terms=13) #

## S2 - B

summary(mo35<-glmmPQL(cbind(Atten_Threat_LatinProp_Exp,100-Atten_Threat_LatinProp_Exp)~Batch+Treatment+Activity+Curiousity+Sociability+Treatment:Activity+Treatment:Curiousity+Treatment:Sociability,random=~1|Group, family=quasibinomial, data=data))

wald.test(vcov(mo35),fixef(mo35), Terms=8) #

wald.test(vcov(mo35),fixef(mo35), Terms=9) #

wald.test(vcov(mo35),fixef(mo35), Terms=10) #

## S3 MODEL FINAL

summary(mo35_f<-glmmPQL(cbind(Atten_Threat_LatinProp_Exp,100-Atten_Threat_LatinProp_Exp)~Batch+Treatment+Activity+Curiousity+Sociability+Treatment:Activity+Treatment:Sociability,random=~1|Group, family=quasibinomial, data=data))

wald.test(vcov(mo35_f),fixef(mo35_f), Terms=1:3)

wald.test(vcov(mo35_f),fixef(mo35_f), Terms=4)

wald.test(vcov(mo35_f),fixef(mo35_f), Terms=5)

wald.test(vcov(mo35_f),fixef(mo35_f), Terms=6)

wald.test(vcov(mo35_f),fixef(mo35_f), Terms=7)

wald.test(vcov(mo35_f),fixef(mo35_f), Terms=8) # s Treatment:Activity

wald.test(vcov(mo35_f),fixef(mo35_f), Terms=9) # s Treatment:Sociability

model_obj_em<-emmeans(mo35_f, ~ Treatment:Activity)

summary(pairs(model_obj_em),adjust="none") # *4 for Bonf correction

# contrast estimate SE df t.ratio p.value

# neg,Act - pos,Act 1.052 0.647 8 1.626 0.1426

# neg,Act - neg,Inact -2.525 0.760 19 -3.322 0.0036

# pos,Act - pos,Inact 0.647 0.755 19 0.857 0.4019

# neg,Inact - pos,Inact 4.224 0.804 8 5.256 0.0008

model_obj_em<-emmeans(mo35_f, ~ Treatment:Sociability)

summary(pairs(model_obj_em),adjust="none")

# contrast estimate SE df t.ratio p.value

# neg,Nonsoc - pos,Nonsoc 3.827 0.739 8 5.179 0.0008

# neg,Nonsoc - neg,Soc 2.514 0.739 19 3.404 0.0030

# pos,Nonsoc - pos,Soc 0.135 0.799 19 0.169 0.8676

# neg,Soc - pos,Soc 1.448 0.727 8 1.993 0.0814

# Latency to eat

##S1

summary(mo37<-glmmPQL(cbind(Atten_Feeding_LatinProp_Exp,100-Atten_Feeding_LatinProp_Exp)~Batch+Treatment+Activity+Curiousity+Sociability+Treatment:Activity+Treatment:Curiousity+Treatment:Sociability+Activity:Curiousity+Activity:Sociability+Curiousity:Sociability+Treatment:Activity:Curiousity+Treatment:Curiousity:Sociability,random=~1|Group, family=quasibinomial, data=data))

wald.test(vcov(mo37),fixef(mo37), Terms=14) #

wald.test(vcov(mo37),fixef(mo37), Terms=15) #

##S2

summary(mo37<-glmmPQL(cbind(Atten_Feeding_LatinProp_Exp,100-Atten_Feeding_LatinProp_Exp)~Batch+Treatment+Activity+Curiousity+Sociability+Treatment:Activity+Treatment:Curiousity+Treatment:Sociability+Activity:Curiousity+Activity:Sociability+Curiousity:Sociability,random=~1|Group, family=quasibinomial, data=data))

wald.test(vcov(mo37),fixef(mo37), Terms=11) #

wald.test(vcov(mo37),fixef(mo37), Terms=12) #

wald.test(vcov(mo37),fixef(mo37), Terms=13) #

## S2-B

summary(mo37<-glmmPQL(cbind(Atten_Feeding_LatinProp_Exp,100-Atten_Feeding_LatinProp_Exp)~Batch+Treatment+Activity+Curiousity+Sociability+Treatment:Activity+Treatment:Curiousity+Treatment:Sociability+Curiousity:Sociability,random=~1|Group, family=quasibinomial, data=data))

wald.test(vcov(mo37),fixef(mo37), Terms=8) #

wald.test(vcov(mo37),fixef(mo37), Terms=9) #

wald.test(vcov(mo37),fixef(mo37), Terms=10) #

wald.test(vcov(mo37),fixef(mo37), Terms=11) #

##S3

summary(mo37<-glmmPQL(cbind(Atten_Feeding_LatinProp_Exp,100-Atten_Feeding_LatinProp_Exp)~Batch+Treatment+Activity+Curiousity+Sociability+Treatment:Activity+Treatment:Curiousity+Treatment:Sociability,random=~1|Group, family=quasibinomial, data=data))

wald.test(vcov(mo37),fixef(mo37), Terms=8) #

wald.test(vcov(mo37),fixef(mo37), Terms=9) #

wald.test(vcov(mo37),fixef(mo37), Terms=10) #

##S4 MODEL FINAL

summary(mo37<-glmmPQL(cbind(Atten_Feeding_LatinProp_Exp,100-Atten_Feeding_LatinProp_Exp)~Batch+Treatment+Activity+Curiousity+Sociability,random=~1|Group, family=quasibinomial, data=data))

wald.test(vcov(mo37),fixef(mo37), Terms=1:3)

wald.test(vcov(mo37),fixef(mo37), Terms=4)

wald.test(vcov(mo37),fixef(mo37), Terms=5)

wald.test(vcov(mo37),fixef(mo37), Terms=6) # tendency cur

wald.test(vcov(mo37),fixef(mo37), Terms=7)

# Time spent in locomotion

##S1

summary(mo37<-glmmPQL(cbind(Loc_Prop_Exp,100-Loc_Prop_Exp)~Batch+Treatment+Activity+Curiousity+Sociability+Treatment:Activity+Treatment:Curiousity+Treatment:Sociability+Activity:Curiousity+Activity:Sociability+Curiousity:Sociability+Treatment:Activity:Curiousity+Treatment:Curiousity:Sociability,random=~1|Group, family=quasibinomial, data=data))

wald.test(vcov(mo37),fixef(mo37), Terms=14) #

wald.test(vcov(mo37),fixef(mo37), Terms=15) #

##S2

summary(mo37<-glmmPQL(cbind(Loc_Prop_Exp,100-Loc_Prop_Exp)~Batch+Treatment+Activity+Curiousity+Sociability+Treatment:Activity+Treatment:Curiousity+Treatment:Sociability+Activity:Curiousity+Activity:Sociability+Curiousity:Sociability,random=~1|Group, family=quasibinomial, data=data))

wald.test(vcov(mo37),fixef(mo37), Terms=11) #

wald.test(vcov(mo37),fixef(mo37), Terms=12) #

wald.test(vcov(mo37),fixef(mo37), Terms=13) #

##S3

summary(mo37<-glmmPQL(cbind(Loc_Prop_Exp,100-Loc_Prop_Exp)~Batch+Treatment+Activity+Curiousity+Sociability+Treatment:Activity+Treatment:Curiousity+Treatment:Sociability,random=~1|Group, family=quasibinomial, data=data))

wald.test(vcov(mo37),fixef(mo37), Terms=8) #

wald.test(vcov(mo37),fixef(mo37), Terms=9) #

wald.test(vcov(mo37),fixef(mo37), Terms=10) #

##S4 FINAL

summary(mo37<-glmmPQL(cbind(Loc_Prop_Exp,100-Loc_Prop_Exp)~Batch+Treatment+Activity+Curiousity+Sociability,random=~1|Group, family=quasibinomial, data=data))

wald.test(vcov(mo37),fixef(mo37), Terms=1:3)

wald.test(vcov(mo37),fixef(mo37), Terms=4)

wald.test(vcov(mo37),fixef(mo37), Terms=5)

wald.test(vcov(mo37),fixef(mo37), Terms=6)

wald.test(vcov(mo37),fixef(mo37), Terms=7)

# Time spent in contact with the walls

##S1

summary(mo37<-glmmPQL(cbind(Expl_Arena_Prop_Exp,100-Expl_Arena_Prop_Exp)~Batch+Treatment+Activity+Curiousity+Sociability+Treatment:Activity+Treatment:Curiousity+Treatment:Sociability+Activity:Curiousity+Activity:Sociability+Curiousity:Sociability+Treatment:Activity:Curiousity+Treatment:Curiousity:Sociability,random=~1|Group, family=quasibinomial, data=data))

wald.test(vcov(mo37),fixef(mo37), Terms=14) #

wald.test(vcov(mo37),fixef(mo37), Terms=15) #

##S2

summary(mo37<-glmmPQL(cbind(Expl_Arena_Prop_Exp,100-Expl_Arena_Prop_Exp)~Batch+Treatment+Activity+Curiousity+Sociability+Treatment:Activity+Treatment:Curiousity+Treatment:Sociability+Activity:Curiousity+Activity:Sociability+Curiousity:Sociability,random=~1|Group, family=quasibinomial, data=data))

wald.test(vcov(mo37),fixef(mo37), Terms=11) #

wald.test(vcov(mo37),fixef(mo37), Terms=12) #

wald.test(vcov(mo37),fixef(mo37), Terms=13) #

##S3

summary(mo37<-glmmPQL(cbind(Expl_Arena_Prop_Exp,100-Expl_Arena_Prop_Exp)~Batch+Treatment+Activity+Curiousity+Sociability+Treatment:Activity+Treatment:Curiousity+Treatment:Sociability,random=~1|Group, family=quasibinomial, data=data))

wald.test(vcov(mo37),fixef(mo37), Terms=8) #

wald.test(vcov(mo37),fixef(mo37), Terms=9) #

wald.test(vcov(mo37),fixef(mo37), Terms=10) #

##S4 FINAL

summary(mo37<-glmmPQL(cbind(Expl_Arena_Prop_Exp,100-Expl_Arena_Prop_Exp)~Batch+Treatment+Activity+Curiousity+Sociability,random=~1|Group, family=quasibinomial, data=data)) # ns

wald.test(vcov(mo37),fixef(mo37), Terms=1:3)

wald.test(vcov(mo37),fixef(mo37), Terms=4)

wald.test(vcov(mo37),fixef(mo37), Terms=5)

wald.test(vcov(mo37),fixef(mo37), Terms=6)

wald.test(vcov(mo37),fixef(mo37), Terms=7)

# Time spent with head up

#S1

summary(mo29<-glmmPQL(cbind(Vigi_Prop_Exp,100-Vigi_Prop_Exp)~Batch+Treatment+Activity+Curiousity+Sociability+Treatment:Activity+Treatment:Curiousity+Treatment:Sociability+Activity:Curiousity+Activity:Sociability+Curiousity:Sociability+Treatment:Activity:Curiousity+Treatment:Curiousity:Sociability,random=~1|Group, family=quasibinomial, data=data))

wald.test(vcov(mo29),fixef(mo29), Terms=14) #

wald.test(vcov(mo29),fixef(mo29), Terms=15) #

#S2

summary(mo29<-glmmPQL(cbind(Vigi_Prop_Exp,100-Vigi_Prop_Exp)~Batch+Treatment+Activity+Curiousity+Sociability+Treatment:Activity+Treatment:Curiousity+Treatment:Sociability+Activity:Curiousity+Activity:Sociability+Curiousity:Sociability,random=~1|Group, family=quasibinomial, data=data))

wald.test(vcov(mo29),fixef(mo29), Terms=11) #

wald.test(vcov(mo29),fixef(mo29), Terms=12) #

wald.test(vcov(mo29),fixef(mo29), Terms=13) #

#S3

summary(mo29<-glmmPQL(cbind(Vigi_Prop_Exp,100-Vigi_Prop_Exp)~Batch+Treatment+Activity+Curiousity+Sociability+Treatment:Activity+Treatment:Curiousity+Treatment:Sociability,random=~1|Group, family=quasibinomial, data=data))

wald.test(vcov(mo29),fixef(mo29), Terms=8) #

wald.test(vcov(mo29),fixef(mo29), Terms=9) #

wald.test(vcov(mo29),fixef(mo29), Terms=10) #

#S4 FINAL

summary(mo29<-glmmPQL(cbind(Vigi_Prop_Exp,100-Vigi_Prop_Exp)~Batch+Treatment+Activity+Curiousity+Sociability,random=~1|Group, family=quasibinomial, data=data)) # curiousity, sociability and treatment

wald.test(vcov(mo29),fixef(mo29), Terms=1:3) #

wald.test(vcov(mo29),fixef(mo29), Terms=4) #

wald.test(vcov(mo29),fixef(mo29), Terms=5) #

wald.test(vcov(mo29),fixef(mo29), Terms=6) #

wald.test(vcov(mo29),fixef(mo29), Terms=7) #

```

#### **For the supplementary materials: Positive versus Negative housing**

```{r echo=T, results='hide'}

## For active/inactive

### Negative

data2_neg_neg_act<-subset(data,Treatment=="Negative"&Activity=="Act")

data2_neg_neg_inact<-subset(data,Treatment=="Negative"&Activity=="Inact")

cbind(round(apply(data2_neg_neg_act[,c(27,29,20,22,23,26,24,25)],2,mean,na.rm=TRUE),0),round(apply(data2_neg_neg_act[,c(27,29,20,22,23,26,24,25)],2,se),1))#

cbind(round(apply(data2_neg_neg_inact[,c(27,29,20,22,23,26,24,25)],2,mean,na.rm=TRUE),0),round(apply(data2_neg_neg_inact[,c(27,29,20,22,23,26,24,25)],2,se),1))#

### Positive

data2_pos_pos_act<-subset(data,Treatment=="Positive"&Activity=="Act")

data2_pos_pos_inact<-subset(data,Treatment=="Positive"&Activity=="Inact")

cbind(round(apply(data2_pos_pos_act[,c(27,29,20,22,23,26,24,25)],2,mean,na.rm=TRUE),0),round(apply(data2_pos_pos_act[,c(27,29,20,22,23,26,24,25)],2,se),1))#

cbind(round(apply(data2_pos_pos_inact[,c(27,29,20,22,23,26,24,25)],2,mean,na.rm=TRUE),0),round(apply(data2_pos_pos_inact[,c(27,29,20,22,23,26,24,25)],2,se),1))#

## For fearful/non-fearful

### Negative

data2_neg_neg_fear<-subset(data,Treatment=="Negative"&Fearfulness=="Fear")

data2_neg_neg_nonfear<-subset(data,Treatment=="Negative"&Fearfulness=="Nonfear")

cbind(round(apply(data2_neg_neg_fear[,c(27,29,20,22,23,26,24,25)],2,mean,na.rm=TRUE),0),round(apply(data2_neg_neg_fear[,c(27,29,20,22,23,26,24,25)],2,se),1)) #

cbind(round(apply(data2_neg_neg_nonfear[,c(27,29,20,22,23,26,24,25)],2,mean,na.rm=TRUE),0),round(apply(data2_neg_neg_nonfear[,c(27,29,20,22,23,26,24,25)],2,se),1)) #

### Positive

data2_pos_pos_fear<-subset(data,Treatment=="Positive"&Fearfulness=="Fear")

data2_pos_pos_nonfear<-subset(data,Treatment=="Positive"&Fearfulness=="Nonfear")

cbind(round(apply(data2_pos_pos_fear[,c(27,29,20,22,23,26,24,25)],2,mean,na.rm=TRUE),0),round(apply(data2_pos_pos_fear[,c(27,29,20,22,23,26,24,25)],2,se),1))#

cbind(round(apply(data2_pos_pos_nonfear[,c(27,29,20,22,23,26,24,25)],2,mean,na.rm=TRUE),0),round(apply(data2_pos_pos_nonfear[,c(27,29,20,22,23,26,24,25)],2,se),1))#

## For social/non-social

### Negative

data2_neg_neg_soc<-subset(data,Treatment=="Negative"&Sociability=="Soc")

data2_neg_neg_nonsoc<-subset(data,Treatment=="Negative"&Sociability=="Nonsoc")

cbind(round(apply(data2_neg_neg_soc[,c(27,29,20,22,23,26,24,25)],2,mean,na.rm=TRUE),0),round(apply(data2_neg_neg_soc[,c(27,29,20,22,23,26,24,25)],2,se),1))#

cbind(round(apply(data2_neg_neg_nonsoc[,c(27,29,20,22,23,26,24,25)],2,mean,na.rm=TRUE),0),round(apply(data2_neg_neg_nonsoc[,c(27,29,20,22,23,26,24,25)],2,se),1)) #

### Positive

data2_pos_pos_soc<-subset(data,Treatment=="Positive"&Sociability=="Soc")

data2_pos_pos_nonsoc<-subset(data,Treatment=="Positive"&Sociability=="Nonsoc")

cbind(round(apply(data2_pos_pos_soc[,c(27,29,20,22,23,26,24,25)],2,mean,na.rm=TRUE),0),round(apply(data2_pos_pos_soc[,c(27,29,20,22,23,26,24,25)],2,se),1))#

cbind(round(apply(data2_pos_pos_nonsoc[,c(27,29,20,22,23,26,24,25)],2,mean,na.rm=TRUE),0),round(apply(data2_pos_pos_nonsoc[,c(27,29,20,22,23,26,24,25)],2,se),1))#

```

# CORRELATION ANALYSES

**Spearman correlations on the raw data in the reference conditions**

```{r echo=T, results='hide'}

data_conv<-read.table("/Users/louise/Documents/PhD/Papers/JBT-PT/Rebuttal 1/JBT_ABT_Cor_Ref.txt",h=T)

str(data_conv)

nrow(data_conv)

cor.test(data_conv$ABT_Threat_Prop,data_conv$Prop_Average, method="spearman") # ns

cor.test(data_conv$ABT_Feeding_Prop,data_conv$Prop_Average,method="spearman") # ns

cor.test(data_conv$ABT_Positive_Diff_Att,data_conv$Prop_Average,method="spearman") # ns

cor.test(data_conv$ABT_Threat_Lat,data_conv$Prop_Average,method="spearman") # ns

cor.test(data_conv$ABT_Feed_Lat,data_conv$Prop_Average,method="spearman") # ns

cor.test(data_conv$ABT_Loc,data_conv$Prop_Average,method="spearman") # ns

cor.test(data_conv$ABT_Expl_Arena,data_conv$Prop_Average,method="spearman") # ns

cor.test(data_conv$ABT_Vigi ,data_conv$Prop_Average,method="spearman") # ns

```

**Spearman correlations on the residuals in the experimental conditions**

```{r echo=T, results='hide'}

data<-read.table("/Users/louise/Documents/PhD/Papers/JBT-PT/Rebuttal 1/JBT_ABT_Res.txt",h=T)

str(data)

nrow(data)

## on the residuals to account for treatment difference

cor.test(data$res_ABT_Threat_Prop,data$res_JBT, method="spearman") # 0.803

cor.test(data$res_ABT_Feeding_Prop,data$res_JBT,method="spearman") # 0.409

cor.test(data$res_ABT_Positive_Diff_Att,data$res_JBT,method="spearman") # 0.439

cor.test(data$res_ABT_Threat_Lat,data$res_JBT,method="spearman") # 0.719

cor.test(data$res_ABT_Feed_Lat,data$res_JBT,method="spearman") # 0.429

cor.test(data$res_ABT_Loc,data$res_JBT,method="spearman") # 0.758

cor.test(data$res_ABT_Expl_Arena,data$res_JBT,method="spearman") # 0.645

cor.test(data$res_ABT_Vigi ,data$res_JBT,method="spearman") # 0.906

```
