# Supplementary material for: Housing and personality effects on judgement and attention biases in dairy cows
Source: Sci Rep. 2021 Nov 26;11:22984. doi: 10.1038/s41598-021-01843-w (PMC8626508; doi:10.1038/s41598-021-01843-w)
Supplement: Supplementary file 3 — Supplementary Tables. [file 41598_2021_1843_MOESM3_ESM.pdf]

Housing and personality effects on judgement and attention biases in dairy cows

Louise Kremer[1,2*], Jacinta D. Bus[1], Laura E. Webb[1], Eddie A. M. Bokkers[1], Bas Engel[3], Jozef T.N. van der Werf[2], Sabine K. Schnabel[3], Cornelis G. van Reenen[1,2]

[1] Animal Production Systems Group, Wageningen University & Research, Wageningen, the Netherlands

[2] Livestock Research, Wageningen University & Research, Wageningen, the Netherlands

[3] Biometris, Wageningen University & Research, Wageningen, the Netherlands

* Corresponding author. E-mail: louise.kremer@wur.nl

Supplementary Tables

**Response variables**	**Explanatory variables**								
In the Judgement Bias Task	Hous	Act	Fear	Soc	Hous:Act	Hous:Fear	Hous:Soc	Act:Soc	Fear:Soc
Average latencies to reach the ambiguous cues	-	-	-	-	-	**0.007**	-	**0.004**	-
In the Attention bias task									
Latency to look at the threat	-	-	0.140	-	**<0.001**	-	**0.013**	-	-
Latency to eat	0.790	0.430	0.084	0.530	-	-	-	-	-
Time spent looking at the threat	-	-	-	-	**0.035**	-	-	**-**	**0.011**
Time spent eating	0.084	0.170	0.075	0.100	-	-	-	-	-
Relative positive attention	0.620	0.066	**0.002**	**0.033**	-	-	-	-	-
Time spent in locomotion	**0.024**	0.380	0.058	0.075	-	-	-	-	-
Time spent with head up	**0.005**	0.062	**0.003**	**0.003**	-	-	-	-	-
Time spent in contact with the walls	0.490	0.420	0.880	0.980	-	-	-	-	-

Supplementary Table 1. Effects of Personality and Housing on heifers' responses to the Judgement and Attention Bias Tasks. Hous: Housing, Act: Activity, Fear: Fearfulness, Soc: Sociability. Significant values ($p < 0.05$) are written in bold. Dashes for two-way interactions indicate that the interaction was dropped from the model ($p > 0.10$). Dashes for main effects indicate that the respective factor is engaged in an interaction.

Personality		Latency to look at the threat		Latency to eat from the bucket	
Trait	Class	Reference	Positive	Reference	Positive
Activity	Active	2 ± 1.0	1 ± 0.4	58 ± 12.3	31 ± 14.0
	Inactive	3 ± 0.6	1 ± 0.4	67 ± 11.3	40 ± 11.1
Fearfulness	Fearful	2 ± 0.6	1 ± 0.5	69 ± 11.3	50 ± 12.0
	Non-fearful	3 ± 1.0	1 ± 0.4	56 ± 12.1	24 ± 11.5
Sociability	Social	1 ± 0.4	1 ± 0.4	69 ± 11.6	38 ± 15.6
	Non-social	3 ± 0.9	2 ± 0.4	58 ± 11.6	35 ± 10.0

Personality		Time spent looking at the threat		Time spent eating from the bucket		Relative positive attention	
Trait	Class	Reference	Positive	Reference	Positive	Reference	Positive
Activity	Active	16 ± 4.8	5 ± 1.4	18 ± 7.6	34 ± 11.3	50 ±11.0	80 ± 6.7
	Inactive	17 ± 4.7	7 ± 2.4	12 ± 5.8	38 ± 9.6	46 ± 10.3	76 ± 8.5
Fearfulness	Fearful	21 ± 5.4	8 ± 2.8	8 ± 5.1	25 ± 7.7	33 ± 8.8	66 ± 9.9
	Non-fearful	10 ± 2.8	5 ± 1.5	23 ±7.5	46 ± 11.3	64 ± 10.4	88 ± 4.0
Sociability	Social	13 ± 3.0	5 ± 1.7	14 ± 6.7	30 ± 11.1	55 ± 9.9	74 ± 10.2
	Non-social	19 ± 5.3	7 ± 2.4	16 ± 6.6	41 ± 9.6	42 ± 10.6	80 ± 6.3

Personality		Head up		In locomotion		In contact with walls/floor	
Trait	Class	Reference	Positive	Reference	Positive	Reference	Positive
Activity	Active	30 ± 7.6	22 ± 5.7	23 ± 4.1	21 ± 3.8	13 ± 2.3	11 ± 4.1
	Inactive	35 ± 7.0	19 ± 7.7	19 ± 2.2	16 ± 2.8	13 ± 3.9	14 ± 4.8
Fearfulness	Fearful	38 ± 6.4	28 ± 9.5	23 ± 3.7	22 ± 3.7	11 ± 2.4	13 ± 3.3
	Non-fearful	27 ± 7.8	13 ± 4.2	19 ± 2.3	15 ± 2.4	16 ± 4.0	13 ± 5.8
Sociability	Social	23 ± 6.5	27 ± 12.3	22 ± 4.2	16 ± 3.5	19 ± 3.9	14 ± 7.8
	Non-social	40 ± 6.8	16 ± 4.2	20 ± 2.5	20 ± 3.1	8 ± 1.9	13 ± 3.1

Supplementary Table 2. Behavioural measures obtained in the attention bias test under the reference and the positive conditions *only* for heifers eventually housed under the positive conditions. Results are presented according to personality traits and classes of personality trait. Results are expressed in proportion of trial duration (120 s), except for *Relative positive attention* that is expressed in proportion of heifer's total time spent at looking at the stimuli.

Personality		Latency to look at the threat		Latency to eat from the bucket	
Trait	Class	Reference	Negative	Reference	Negative
Activity	Active	1 ± 0.6	6 ± 2.4	56 ± 11.9	38 ± 11.9
	Inactive	2 ± 1.1	13 ± 5.5	45 ± 16.5	15 ± 9.5
Fearfulness	Fearful	2 ± 0.6	7 ± 3.0	58 ± 12.3	37 ± 14.8
	Non-fearful	2 ± 1.0	8 ± 3.7	45 ± 14.9	26 ± 11.6
Sociability	Social	2 ± 0.7	6 ± 2.7	58 ± 11.9	28 ± 11.5
	Non-social	2 ± 0.9	10 ± 4.6	40 ± 16.3	36 ± 15.8

Personality		Time spent looking at the threat		Time spent eating from the bucket		Relative positive attention	
Trait	Class	Reference	Negative	Reference	Negative	Reference	Negative
Activity	Active	11 ± 2.2	11 ± 4.3	21 ± 9.0	44 ± 10.6	55 ± 8.9	70 ± 10.7
	Inactive	9 ± 3.7	3 ± 0.4	40 ± 14.9	63 ± 11.0	62 ± 16.0	96 ± 0.5
Fearfulness	Fearful	11 ± 2.7	9 ± 2.7	10 ± 4.9	45 ± 13.8	51 ± 8.8	70 ± 12.7
	Non-fearful	9 ± 2.8	9 ± 5.7	46 ± 13.4	53 ± 10.2	64 ± 13.8	85 ± 10.3
Sociability	Social	13 ± 2.6	10 ± 4.8	23 ± 8.7	47 ± 10.0	50 ± 11.1	75 ± 11.5
	Non-social	6 ± 1.8	6 ± 2.1	38 ± 16.9	53 ± 15.7	73 ± 8.9	81 ± 10.1

Personality		Head up		In locomotion		In contact with walls/floor	
Trait	Class	Reference	Negative	Reference	Negative	Reference	Negative
Activity	Active	18 ± 6.0	13 ± 4.3	31 ± 5.0	13 ± 2.7	17 ± 3.2	10 ± 2.6
	Inactive	15 ± 5.6	7 ± 4.3	14 ± 3.8	13 ± 2.2	6 ± 1.9	11 ± 5.2
Fearfulness	Fearful	25 ± 6.9	15 ± 6.2	30 ± 5.5	13 ± 3.7	14 ± 3.3	8 ± 3.1
	Non-fearful	9 ± 4.3	8 ± 2.9	18 ± 4.7	12 ± 2.0	12 ± 3.8	11 ± 3.4
Sociability	Social	16 ± 3.6	13 ± 4.5	22 ± 4.4	13 ± 2.3	13 ± 3.0	11 ± 3.1
	Non-social	18 ± 10.3	8 ± 4.6	28 ± 7.6	11 ± 4.0	12 ± 4.7	8 ± 3.6

Supplementary Table 3. Behavioural measures obtained in the attention bias test under the reference and the negative conditions *only* for heifers eventually housed under the negative conditions. Results are presented according to personality traits and classes of personality trait. Results are expressed in proportion of trial duration (120 s), except for *Relative positive attention* that is expressed in proportion of heifer's total time spent at looking at the stimuli.

Personality		Latency to look at the threat		Latency to eat from the bucket	
Trait	Class	Positive	Negative	Positive	Negative
Activity	Active	1 ± 0.4	6 ± 2.4	31 ± 14.0	38 ± 11.9
	Inactive	1 ± 0.4	13 ± 5.5	40 ± 11.1	15 ± 9.5
Fearfulness	Fearful	1 ± 0.5	7 ± 3.0	50 ± 12.0	37 ± 14.8
	Non-fearful	1 ± 0.4	8 ± 3.7	24 ± 11.5	26 ± 11.6
Sociability	Social	1 ± 0.4	6 ± 2.7	38 ± 15.6	28 ± 11.5
	Non-social	2 ± 0.4	10 ± 4.6	35 ± 10.0	36 ± 15.8

Personality		Time spent looking at the threat		Time spent eating from the bucket		Relative positive attention	
Trait	Class	Positive	Negative	Positive	Negative	Positive	Negative
Activity	Active	5 ± 1.4	11 ± 4.3	34 ± 11.3	44 ± 10.6	80 ± 6.7	70 ± 10.7
	Inactive	7 ± 2.4	3 ± 0.4	38 ± 9.6	63 ± 11.0	76 ± 8.5	96 ± 0.5
Fearfulness	Fearful	8 ± 2.8	9 ± 2.7	25 ± 7.7	45 ± 13.8	66 ± 9.9	70 ± 12.7
	Non-fearful	5 ± 1.5	9 ± 5.7	46 ± 11.3	53 ± 10.2	88 ± 4.0	85 ± 10.3
Sociability	Social	5 ± 1.7	10 ± 4.8	30 ± 11.1	47 ± 10.0	74 ± 10.2	75 ± 11.5
	Non-social	7 ± 2.4	6 ± 2.1	41 ± 9.6	53 ± 15.7	80 ± 6.3	81 ± 10.1

Personality		Head up		In locomotion		In contact with walls/floor	
Trait	Class	Positive	Negative	Positive	Negative	Positive	Negative
Activity	Active	22 ± 5.7	13 ± 4.3	21 ± 3.8	13 ± 2.7	11 ± 4.1	10 ± 2.6
	Inactive	19 ± 7.7	7 ± 4.3	16 ± 2.8	13 ± 2.2	14 ± 4.8	11 ± 5.2
Fearfulness	Fearful	28 ± 9.5	15 ± 6.2	22 ± 3.7	13 ± 3.7	13 ± 3.3	8 ± 3.1
	Non-fearful	13 ± 4.2	8 ± 2.9	15 ± 2.4	12 ± 2.0	13 ± 5.8	11 ± 3.4
Sociability	Social	27 ± 12.3	13 ± 4.5	16 ± 3.5	13 ± 2.3	14 ± 7.8	11 ± 3.1
	Non-social	16 ± 4.2	8 ± 4.6	20 ± 3.1	11 ± 4.0	13 ± 3.1	8 ± 3.6

Supplementary Table 4. Behavioural measures obtained in the attention bias test under the positive and the negative conditions. Results are presented according to personality traits and classes of personality trait. Results are expressed in proportion of trial duration (120 s), except for *Relative positive attention* that is expressed in proportion of heifer's total time spent at looking at the stimuli.
